# Nano-engineering nanomedicines with customized functions for tumor treatment applications

**DOI:** 10.1186/s12951-023-01975-3

**Published:** 2023-08-02

**Authors:** Yuxin Wang, Shimei Li, Xiangling Ren, Shiping Yu, Xianwei Meng

**Affiliations:** 1 Laboratory of Controllable Preparation and Application of Nanomaterials, Technical Institute of Physics and Chemistry, Chinese Academy of Sciences, Beijing, 100190 China; 2 CAS Key Laboratory of Cryogenics, Technical Institute of Physics and Chemistry, Chinese Academy of Sciences, Beijing, 100190 China; 3 University of Chinese Academy of Sciences, 100049 Beijing, China; 4 Shanxi Province Cancer Hospital/Shanxi Hospital Affiliated to Cancer Hospital, Chinese Academy of Medical Sciences/Cancer Hospital Affiliated to Shanxi Medical University, Taiyuan, 030013 China

**Keywords:** Nano-engineering, Nanomedicines, Function customization, Tumor treatment

## Abstract

Nano-engineering with unique “custom function” capability has shown great potential in solving technical difficulties of nanomaterials in tumor treatment. Through tuning the size and surface properties controllablly, nanoparticles can be endoewd with tailored structure, and then the characteristic functions to improve the therapeutic effect of nanomedicines. Based on nano-engineering, many have been carried out to advance nano-engineering nanomedicine. In this review, the main research related to cancer therapy attached to the development of nanoengineering nanomedicines has been presented as follows. Firstly, therapeutic agents that target to tumor area can exert the therapeutic effect effectively. Secondly, drug resistance of tumor cells can be overcome to enhance the efficacy. Thirdly, remodeling the immunosuppressive microenvironment makes the therapeutic agents work with the autoimmune system to eliminate the primary tumor and then prevent tumor recurrence and metastasis. Finally, the development prospects of nano-engineering nanomedicine are also outlined.

## Introduction

According to a report released by the World Health Organization in 2020, there were 19.3 million new cancer cases and 1 million cancer deaths worldwide, and the incidence and mortality of cancer were both showing an upward trend, seriously threatening human health [1–4]. At present, great progress has been made in the research of tumor treatment [5], but the therapeutic effect of nanomedicines is still limited by some problems, such as the poor delivery [6–9], the drug resistance of tumor cells [10–12], the immunosuppressive microenvironment [13–16] and so on.

In recent years, the development of nanotechnology has provided many new research ideas for tumor treatment, especially the proposal of nano-engineering nanomedicines. Nano-engineering refers to the adjustment of size, surface properties and structure of nanoparticles. Based on nano-engineering, (1) the size can be controlled by adjusting synthesis conditions, and then the circulation, distribution, and excretion of nanomedicines in the body can be improved; (2) the surface properties can be changeed through physical or chemical methods to improve the dispersion, stability, biocompatibility and surface reactivity of nanomedicines; (3) the structure of nanomedicines can be precisely designed to regulate their overall performance. Therefore, nano-engineering can carry out “customized functions” for specific nanomedicines, playing an extremely important role in tumor treatment.

Conventional delivery methods have poor pharmacokinetics and non-specific toxicity [17], which not only have great toxicity and side effects on normal tissues, but also weaken the effect of tumor treatment. Nanocarriers with customized functions based on nano-engineering can prolong the half-life of blood circulation, improve tumor specificity, and effectively accumulate in the tumor area, thus enhancing the therapeutic effect. Drug resistance refers to the resistance of tumor cells to therapeutic agents, resulting in poor effect and ultimately affecting the therapeutic effects [10, 18, 19]. Nano-engineering nanomedicines can effectively inhibit drug resistance by increasing intracellular therapeutic agent accumulation, thus improving tumor treatment effects. In addition, a large number of immunosuppressive cells and immunosuppressive cytokines in the tumor microenvironment, as well as the loss of immunogenicity of tumor cells and immune checkpoints, will suppress the immune response [13, 14]. Nano-engineering nanomedicines with customized functions can effectively alleviate immunosuppression and enhance immune response.

**Scheme 1:**
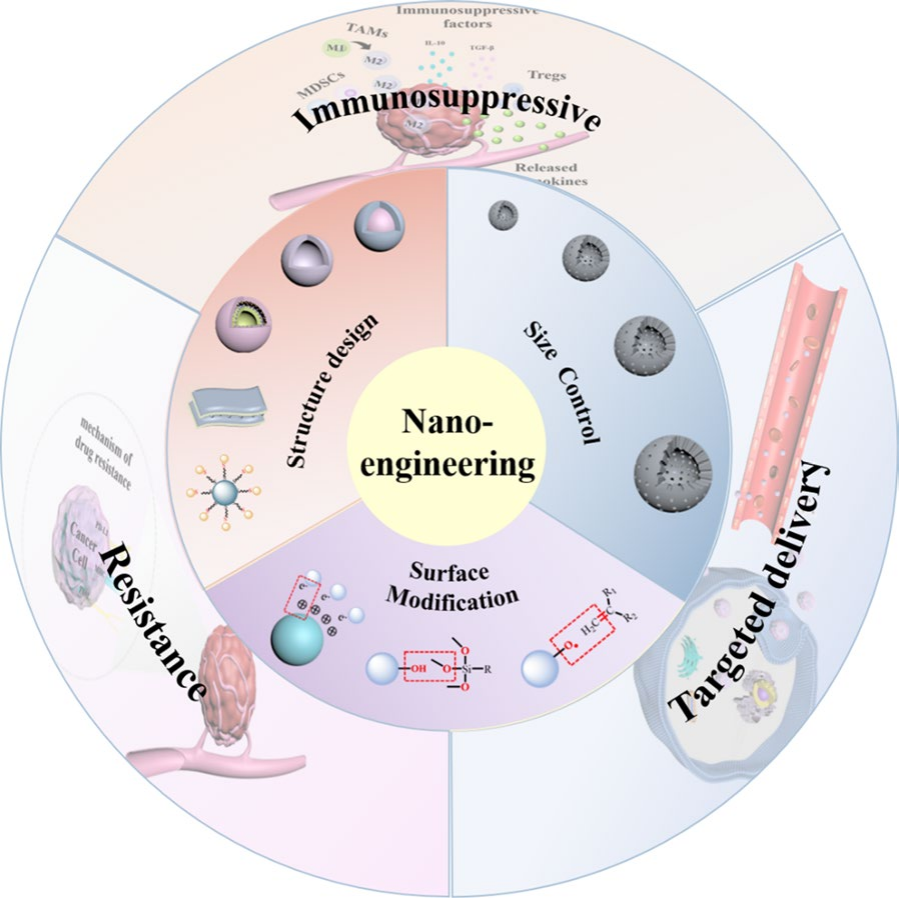
A brief introduction of nano-engineering and their vital applications in tumor treatment

To better understand the application of nano-engineering nanomedicines in cancer therapy, this review firstly introduced the relevant contents of size control, surface modification and structure design from the perspective of nano-engineering. Then, it summarized the applications of nano-engineering for obtaining nanomedicines with customized functions to overcome their deficiency in tumor treatments, including the poor delivery, tumor drug resistance, and the immunosuppressive microenvironment (Scheme 1). Finally, the prospects and challenges of nano-engineering nanomedicines in promoting the development of cancer therapeutics have also been discussed.

## Design strategies of nano-engineering

### Controlling the size

Due to the surface effects, quantum size effects and macroscopic quantum tunneling effects of nanomaterials, the properties of nanoparticles are significantly dependent on their size, such as acoustic, optical, electrical, magnetic, thermal, mechanical, and chemical catalysis [20]. In addition, size also affects its blood circulation life, distribution and excretion in the body [21]. Therefore, controlling the size of nanoparticles is a very effective way to control the properties of nanoparticles. This section briefly introduces nano-engineering to control the size of nanoparticles, and Table 1 summarizes the parameters that affect the size of nanoparticles.

**Fig. 1:**
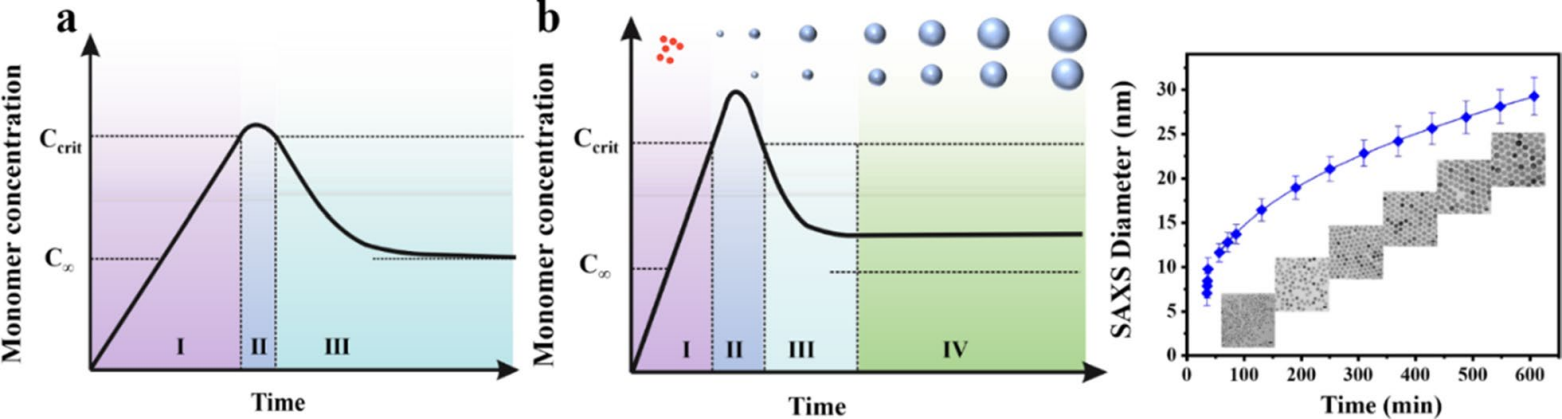
**a** Schematic diagram of LaMer mechanism. I: the monomer formation stage, II: the nucleation stage, III:the growth stage. **b** Schematic illustration of the time-dependent change in size of magnetite nanoparticles synthesized based on the extended LaMer mechanism

To control the size of nanoparticles, it is first necessary to understand their growth mechanism. The most common growth mechanism is the LaMer mechanism (Fig. 1a). The LaMer mechanism is divided into three stages: The first is the monomer formation stage, where the concentration of free monomer in the precursor decomposition solution increases rapidly; then there is the nucleation stage, the monomer concentration is higher than the critical nucleation concentration, monomer burst nucleation causes the concentration of free monomer in the solution to drop rapidly, and the nucleation is terminated; the last is the growth stage, where the monomer diffuses to the surface of the core to grow [22–25]. According to the LaMer mechanism, it is not difficult to conclude that by changing the concentration of reactants, solvent ratio, reaction temperature, and stabilizers, the growth of nanoparticles can be controlled to control the size.

Controlling the ratio of reducing agent to precursor is an important method for changing the size of nanoparticles. It can be explained by the LaMer mechanism: the reducing agent reduces the precursor to form monomer, and controlling the ratio of the reducing agent to the precursor is equivalent to controlling the concentration of the monomer initially used for nucleation. When the ratio of the reducing agent to the precursor is smaller, the fewer seeds are formed in the initial nucleation stage, and the monomers obtained later are used for growth instead of nucleation, so the final size of the nanoparticles obtained is larger. Laura Hippolyte et al. controlled nucleation and growth of Au nanoparticles (NPs) by changing the ratio of NHC-BH_3_ and Au precursors. When the ratio of NHC-BH_3_ to Au precursor is 1:1, the size of Au NPs is 10.0 ± 2.7 nm. With the increase of the ratio of NHC-BH_3_, the size of Au NPs decreases, and when the ratio is 28:1, the size decreased to 4.9 ± 0.9 nm [26]. Minh Tran and colleagues used citric acid to reduce Au to verify that the size of Au nanoparticles depends on the ratio of Na_3_Ct/HAuCl_4_ (Fig. 1c) [27]. Yeojin Jeon and his colleagues used FeCl_3_ and palmitic acid to synthesize Fe_3_O_4_ nanoparticles, when the mixing ratio of FeCl_3_ and palmitic acid is 1:1, the size of the nanoparticles is about 800 nm, the larger the ratio of palmitic acid, the smaller the size of the nanoparticles. When the ratio is 4:1, the size of the nanoparticles is about 40 nm [28].

The reaction time is also an important parameter to control the size of nanoparticles. When the monomer concentration exceeds the critical nucleation concentration and burst nucleation, the remaining monomer diffuses to the surface of the nucleus to grow. Therefore, before the monomer is completely consumed, the longer the reaction time, the larger the size of the nanoparticles. Florian Mayer and colleagues obtained nanoparticles of different sizes by adjusting the reaction time. They found that after 30, 48, and 60 min of reaction, nanoparticles with increasing sizes were obtained, which were 53.6, 71.1, and 79.8 nm, respectively [29]. Kenya Komoda and Takehiro Kawauchi found that the size of nanoparticles would increase with the extension of reaction time. When the reaction time is increased from 10 to 20 min, the particle size of the NPs increases from 142 ± 15 nm to 269 ± 67 nm [30]. This was also demonstrated by Auni Hamimi Idrisa and his colleagues. When the reaction time was increased from 15 to 60 min, the particle size of the iron oxide NPs increased from 4.9 to 8.6 nm [31]. In addition, if the monomer quantity in the solution can be maintained without allowing it to exceed the critical nucleation concentration again, the nanoparticles continue to grow, and their size is proportional to the reaction time, so that the size of the nanoparticles can be precisely controlled by controlling the reaction time. Erika C. Vreeland et al. continuously added precursors to the reaction system to maintain the nanoparticle concentration, so that the nanoparticles could grow steadily for any length of time. During this process, the particle size increases with the reaction time, and the growth rate of the steady-state growth is constant, and the particle size can be predicted by the reaction time (Fig. 1b) [32].

The effect of reaction temperature on nanoparticle size is complex. First, temperature affects the nucleation stage of nanoparticles, according to the classical nucleation theory, as the reaction temperature increases, the nucleation rate accelerates, thus theoretically obtaining smaller size particles. For example, the Au nanoparticles prepared by Minh Tran et al. decreased in size with increasing temperature (Fig. 1c) [27]. And Yuan et al. showed that as the reaction temperature increased from 993 to 1373 K, the average size of TiB_2_ changed from 22.1 to 171.4 nm [33]. However, in the growth stage, the higher the reaction temperature, the more intense the Brownian motion of the monomer, which increases the probability of the particles contacting each other, and the nanoparticles can continue to grow, resulting in larger-sized nanoparticles. Derrick Mott et al. synthesized highly monodispersed Cu NPs with an average size in the range of 5–25 nanometers in an organic suspension by changing the temperature. Their study proved that the average size of copper nanoparticles increases approximately linearly with temperature [34].

For polymer nanoparticles, their preparation usually requires solvent and non-solvent phases (organic and aqueous phase), which involves complex interfacial hydrodynamic phenomena [35–37]. Two-phase mixing causes the polymer in the organic phase to transfer to the aqueous phase and aggregate to obtain polymer nanoparticles. Good mixing conditions and large polymer concentrations will lead to a local supersaturation to reach the critical nucleation concentration, which leads to rapid and large nucleation, and eventually to smaller nanoparticles [38, 39]. It is not difficult to see that the choice of organic solvent and the initial concentration of the polymer in the organic phase affect the size of the polymer NPs. For example, Moritz Beck-Broichsitter et al. demonstrated that organic solvents with higher water affinity can more effectively promote polymers into the aqueous phase to obtain smaller nanoparticles. And with the increase of polymer concentration, the particle size increases linearly. They used Dimethyl sulfoxide (DMSO) and Tetrahydrofuran (THF) as solvents, respectively, to obtain NPs of 66 and 162 nm at an initial concentration of 10 mg ml^− 1^ and 89 and 223 nm NPs at an initial concentration of 25 mg ml^− 1^[40]. Miechel L.T. Zweers et al. also demonstrated that increasing polymer concentration leads to an increase in particle size. In their study, by varying the concentration of the polymer, monodisperse poly(lactic-co-glycolic acid) (PLGA) nanoparticles of 100–400 nm size could be obtained [41].

The charge of the polymer also affects the particle size. According to classical nucleation theory, the final size of the nanoparticle depends on nucleation and growth, and the more the number of nucleation and the slower the growth rate, the smaller the resulting nanoparticle size. In the nucleation process of particles, the charged groups can reduce the interfacial tension and increase the nucleation rate; At the same time, due to the existence of charge, the charge repulsion reduces the polymer around the nucleus, reducing the growth rate, resulting in smaller nanoparticles. In the study of Andreas Reisch et al., they introduced negatively charged carboxylate and sulfonate as well as positively charged trimethylammonium groups into polymers. The carboxylate group is easily protonated and have less charge. Finally, carboxylate bearing polymers obtained nanoparticles of 60–100 nm, while the polymer containing sulfonate and trimethylammonium obtained nanoparticles smaller than 25 nm [42]. Vitalii Rosiak et al. also demonstrated that by using sulfonate-containing polymers, the size can be further reduced, resulting in nanoparticles of only 10 nm [43].

Stabilizer is an important parameter that affects the size of nanoparticles. Only when the monomer diffuses to the surface of the nanoparticle, the nanoparticle can grow, and the stabilizer controls the size by affecting the surface state of the nanoparticle. First, the stabilizer can directly aggregate on the nanoparticle surface, competing with the aggregation of reactant monomers on the nanoparticle surface; second, the stabilizer can prevent the reactant monomer from approaching the nanoparticle surface through its own steric hindrance; in addition, the stabilizer alters the properties of the nanoparticle surface, such as hydrophobicity and charge, by interacting with the nanoparticle surface, which controls the aggregation of reactant monomers on the nanoparticle surface, thereby controlling the size of the nanoparticle. Stabilizers include polymers, surfactants, metal complexes and others. Kentaro Ichihashi et al. used an organic polymer polypyrrole (PPy) to coat Pt NP, and obtained nanoparticles with a controllable size [44]. Sang-Wook Kim et al. obtained Pd NPs of different sizes by using different surfactants. They found that when trioctylphosphine (TOP) was used as a surfactant and solvent, particles of about 3.5 nm were obtained. When a mixture of TOP and oleylamine is used, the particle size is 5 and 7 nm, and when only oleylamine is used, the particle size is above 10 nm [45]. Ce ´dric R. Mayer et al. used metal complexes as stabilizers to prepare Ag NP. When the complex has a strong interaction with the Ag NP surface, the surface of the nanoparticle is rapidly coated and the growth is stagnated. Therefore, the stronger the interaction between the complex and the Ag NP surface, the smaller the nanoparticle size, and as the silver/ruthenium (ii) ratio increases, the surface coating decreases and the particle size increases [46].

The effect of stabilizers on polymer nanoparticles is more complex. Katsuhiro Onita and his colleagues found that as polyvinylpyrrolidone (PVP) concentration increased from 3 to 8 wt%, the particle size of polymer particles decreased from 1.8 to 1.2 μm [47]. In the study by Yupaporn Niyom et al., the size of the nanoparticles decreased with increasing surfactant concentration [48]. But Benita et al. demonstrated that particle size increases as stabilizer concentration increases [49]. This data seems contradictory, but in fact there are different theoretical supports behind it. When the stabilizer concentration is low, it covers the surface of the droplet, reduces the interfacial tension, prevents aggregation, and obtains nanoparticles with smaller size; When the concentration exceeds the threshold, the increase in concentration cannot further reduce the interfacial tension, and the stabilizer exists in a continuous phase, and the viscosity in the system increases, thereby increasing the particle size. This is evidenced by the study of Miechel L.T. Zweers et al., who used Polyvinyl alcohol (PVA) as a stabilizer, and in the range of 0–2%, the PLGA particle size decreased as PVA concentration increased; In the range of 2–5%, the particle size is almost constant; By further increasing the concentration, the particle size increases [41].

In short, by adjusting the concentration and ratio of reactants, reaction time, temperature, solvent, charge, stabilizer and other parameters, the number of nucleation or the growth state of nanoparticles can be controlled, thereby effectively controlling the size of nanoparticles. Furthermore, in general, the size of nanoparticles is not controlled by a single factor, but by multiple parameters.

**Table 1:** Summary of parameters affecting particle size

Parameter	Effect	Reason
The ratio of reducing agent to precursor	The larger the ratio of reducing agent to precursor, the smaller the size.	More nucleation
Reaction time	The longer the reaction time, the larger the size.	More growth
Temperature	Complex	Temperature affects the nucleation and growth stages of nanoparticles.
Solvent	The larger the ratio of organic solvents, the smaller the size.	More nucleation
Charge	The more charge, the smaller the size.	More nucleation and less growth
Stabilizer	Complex	Stabilizers affect the surface state of nanoparticles.

### Surface modification

Nanoparticles have the inherent characteristics such as large specific surface area, easy to modify their surface, etc., which have broad application prospects in the fields of biomedicine, catalysis, lithium batteries and so on. However, these characteristics also bring certain problems. On the one hand, nanoparticles with large specific surface area have a large surface Gibbs free energy, which is prone to aggregation or oxidation; on the other hand, there are many unsaturated bonds and dangling bonds on the surface of nanoparticles, which are easily combined with other atoms, resulting in poor stability. These factors hinder the practical application of nanomaterials. Appropriate surface modification of nanoparticles can overcome the disadvantages and obtain the desired materials. Surface modification here refers to the use of physical or chemical methods to change the structure or state of the nanoparticle surface, thereby controlling its surface properties. The surface modification methods of nanomaterials mainly include adsorption, grafting and coupling. These methods are briefly introduced below, and Table 2 summarizes the comparison between the four methods.

#### Adsorption

Adsorption includes physical adsorption and chemical adsorption, and is usually attached to the surface of nanoparticles with surfactants. Physical adsorption is non-selective monolayer or multi-molecular layer adsorption through van der Waals forces, while chemical adsorption is monolayer adsorption through electrostatic interaction or hydrogen bonding. There is no clear boundary between physical adsorption and chemical adsorption. The same substance may be physically adsorbed at low temperature and chemically adsorbed at high temperature. Through adsorption, the surface charge and hydrophilicity of nanoparticles can be changed, the interaction between particles can be reduced, and the dispersibility and biocompatibility of nanoparticles can be improved. Ahmed Barhoum et al. used hexadecyl trimethyl ammonium Bromide (CTAB) (cationic surfactant) and sodium oleate (anionic surfactant) to modify the surface of calcium carbonate by adsorption. Their research shows that CTAB, as a quaternary ammonium compound (NR_4_^+^Br^-^), can only interact with CaCO_3_ particles through van der Waals forces, while the carboxyl group (-COO^-^) of sodium oleate can effectively combine with Ca^2+^ on the surface of CaCO_3_ particles through electrostatic interaction. The adsorption capacity of CTAB and oleate are 20% and 75%, respectively. Whether it is surface modification by CTAB or sodium oleate, the surface potential of the nanoparticles is changed, and the dispersion of the particles is effectively improved [50]. Surface modification of nanoparticles by adsorption is one of the easiest ways to improve the performance of nanoparticles. However, because they are fixed on the surface of nanoparticles through relatively weak van der Waals forces or electrostatic interactions and other non-covalent bonds, they are unstable under external stimulation.

#### Coupling

Coupling refers to the combination of two units to form a molecule, which requires the assistance of a coupling agent. Coupling agents generally have two groups, one hydrophilic and the other hydrophobic. Inorganic nanoparticles (high surface energy) and organics (low surface energy) are difficult to be compatible when mixed due to the difference in surface energy. The hydrophobic groups of the coupling agent can chemically react with the surface of inorganic substances, and the hydrophilic groups can react with or be compatible with organic substances. Therefore, the coupling agents can improve the compatibility between inorganic nanoparticles and organic molecules.

**Fig. 2:**
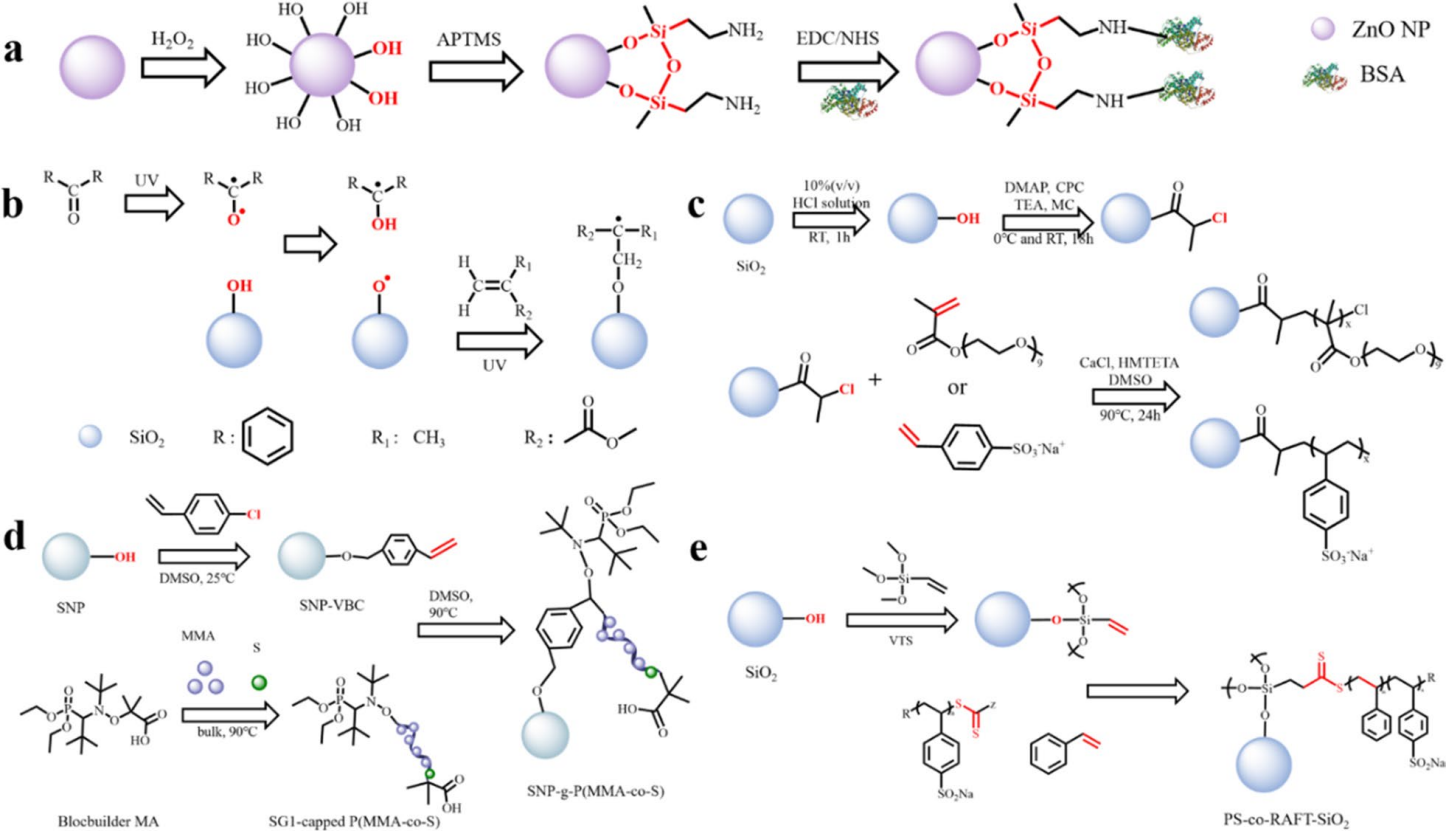
**a** Schematic diagram of the surface modification process of ZnO NPs by coupling method. Schematic illustration of graft polymerization from NPs via **b **UV-induced free radical polymerization; **c** atom transfer radical polymerization; **d** nitrogen oxide mediated polymerization and **e** reversible addition-fragmentation chain transfer polymerization

The most commonly used coupling agent is the silane coupling agent ((R'O)_3_SiR). The silane coupling agent is usually used for inorganic nanoparticles with hydroxyl groups on the surface. The alkoxy group (-OR') condenses with -OH to form a covalent bond to stably modify the surface of the nanoparticle [51]. In addition, the silane coupling agent usually has various other functional groups, which can be used for further modification. Li et al. used KH550 silane coupling agent to modify the surface of TiO_2_ nanoparticles. The -OR' of the silane coupling agent first forms a hydrogen bond with the -OH on the surface of the TiO_2_, and then dehydrates and condenses to form a Si-O-Ti bond, which is stably modified on the surface of the nanoparticle. The hydrophilic group and steric hindrance effect of the silane coupling agent effectively improve the dispersion of nanoparticles [52]. Similarly, Li et al. used silane coupling agent to modify Al_2_O_3_ NPs to improve their dispersion stability [53]. Hong et al. modified the KH-570 coupling agent on the surface of zinc oxide nanoparticles, and then fixed polystyrene (PS) on the surface of the nanoparticles by grafting to improve the dispersibility [54]. Their research results also show that coupling can effectively improve the compatibility of inorganic nanoparticles and organic molecules. A.I. Barabanova and his colleagues used 3-(triethoxysilyl) propylsuccinic anhydride (TESPSA) to modify SiO_2_ nanoparticles. They modified the acid anhydride group to the surface of the nanoparticle through the condensation reaction of TESPSA ethoxy group and -OH group on the surface of SiO_2_. It not only improves the dispersibility of nanoparticles, but also enhances surface activity, so that nanoparticles can further react with other polymers [55]. Seda Kelestemur et al. used a coupling agent to modify bovine serum albumin (BSA) onto the surface of ZnO. They first treated with H_2_O_2_ to increase the -OH density on the surface of ZnO NPs, and then modified the silane coupling agent 3-aminopropyltrimethoxysilane (APTMS) on the surface of ZnO to obtain nanoparticles with amine functional groups on the surface. Finally, the EDC/NHS coupling method was used to modify the BSA to the surface of the nanoparticles through the amide bond, which effectively improved the biocompatibility of the nanoparticles (Fig. 2a) [56]. Therefore, the coupling agent is suitable for the composite of inorganic nanoparticles and organics. After the surface of the inorganic nanoparticles is treated with the coupling agent, it can produce high compatibility with organics, and it is conducive to the mono-dispersion of the nanoparticles. In addition, after the coupling agent is modified, other organics can be further modified on the surface to obtain nanoparticles with desired properties.

#### Grafting

Grafting is a chemical modification method that binds macromolecular chains to the surface of the material through covalent bonds, thereby changing the surface properties of nanoparticles. According to different grafting methods, it can be divided into grafting-to approach and grafting-from approach. The grafting-to approach refers to the direct covalent connection between the polymer and the functional group on the surface of the material. This method can graft high-molecular-weight polymers with low steric hindrance, but the conditions are relatively harsh; The grafting-from approach means that the monomer is directly polymerized in situ on the surface of the material under the action of the initiator, which can accurately control the molecular weight and density of the graft, but in the late stage of the reaction, the steric hindrance is large, and the molecular weight of the grafted polymer is small. In these two grafting methods, the polymer or monomer needs to be activated by physical or chemical methods to initiate the polymerization grafting process. In recent years, conventional free radical polymerization and reversible deactivation radical polymerization (RDRP) have attracted widespread research interest.

In conventional free radical polymerization, photo-initiated grafting is a mature graft modification technology. As early as 1957, Gerald Oster et al. used ultraviolet light to initiate grafting on the polymer surface [57]. Photo-initiated grafting is the use of an initiator to absorb ultraviolet light or visible light to generate free radicals, which are then transferred to the surface of the nanoparticles, and then react with the polymer for grafting. At present, benzophenone is usually used as an ultraviolet light initiator. Sooyeon Kim et al. modified the silica surface by grafting method using methyl methacrylate (MMA) under far-ultraviolet light irradiation. The photo-initiator benzophenone absorbs extreme ultraviolet light (250 nm) to form free radicals, and deprives the hydrogen from -OH on the SiO_2_ surface to form free radicals on the SiO_2_ surface. It reacts with MMA to grow the graft chain, and Si-O-C bond is formed on the surface of SiO_2_. Surface modification improves the stability of nanoparticles, and due to the grafting of active monomers, the surface activity of SiO_2_ is improved so that it can react with other substances (Fig. 2b) [58]. Lou et al. also used benzophenone as an initiator to photopolymerize N-[2-(acryloyloxy) ethyl]-N, N-dimethyl-N-butylammonium iodide on the surface of polydimethylsiloxane, and its high-density quaternary ammonium salt surface can be applied to sterilization [59]. In the study of Radmila Tomovska et al., TiO_2_ nanoparticles, which can directly generate highly active free radicals under photocatalysis, react with silane coupling agent (3-chloropropyl) triethoxysilane (TCPEOS) under light, and are successfully grafted through Ti-O-Si bonds to obtain nanoparticles with chloropropyl functional groups on the surface. In addition, the ethoxy group is hydrolyzed and condensed to form a Si-O-Si network on the surface, so that the surface of the nanoparticle has super-hydrophobicity [60]. However, when TCPEOS is used for grafting, free radicals will break the Si-C bond and disappear the functional groups that have been modified on the surface of the nanoparticles. Therefore, they tried another silane coupling agent, 3-triethoxysilyl propyl isocianate (PIC), under the same reaction conditions as the TCPEOS reaction, the Si-C bond does not crack when using PIC [61]. Their research results show that by changing the terminal functional groups of the grafted polymer, the surface properties of nanoparticles can be effectively controlled. The light-initiated grafting reaction conditions are mild, the reaction process is pollution-free, and the post-reaction treatment is simple, so it has a wide range of applications. In addition to photo-initiation, there are other activation methods, such as plasma initiation [62], radiation initiation [63], and microwave initiation [64], which are not described in detail here.

In 1956, Szwar first proposed the concept of living polymerization [65]. After decades of development and improvement, the theory of RDRP has matured. RDRP methods include atom transfer radical polymerization (ATRP), reversible addition-fragmentation chain transfer polymerization (RAFT) and nitrogen oxide mediated polymerization (NMP) [66]. All of these living radical polymerization methods can control the types and density of grafted polymers on the surface of nanoparticles to achieve precise regulation.

ATRP uses organic halides as initiators. The organic halides are first homogenized to generate alkyl radicals (R·), and then the formed R· undergo chain growth through double bond addition, and chain termination occurs through disproportionation or coupling. ATRP can graft polymers with specific functional groups and molecular weights onto the surface of nanoparticles, thereby tailoring the surface properties of nanoparticles according to the required properties [67]. Jung Tae Park et al. first activated the silanol groups (Si-OH) on the surface of SiO_2_ nanoparticles, and then combined with 2-chloropropionyl chloride (CPC) to convert the surface -OH to -Cl. Finally, using chlorine atom as an initiator, the hydrophilic polymer was grafted onto the SiO_2_ surface through ATPR. Due to the mutual repulsion and steric hindrance between hydrophilic polymers, the dispersion performance of nanoparticles is improved (Fig. 2c) [68]. Guo et al. directly prepared SiO_2_ with -Br, using -Br as an ATRP-initiating group to graft azobenzene polymer brush, and then modified with glycine to obtain a photosensitive nano-adsorbent that can selectively remove low-density lipoprotein [69].

NMP uses alkoxyamine as initiator to generate free radicals for graft polymerization. It can also precisely design and functionalize the surface of nanoparticles. However, because the preparation of alkoxyamines is more complicated and generally only suitable for styrene, there has been less research in recent years. Jaime C. Cazotti and his colleagues used NMP to modify starch nanoparticles (SNP) to improve the dispersion of nanoparticles. First, the SNP is modified with 4-vinylbenzyl chloride (VBC), and the surface is modified with reactive double bonds. Then, SG1 capped polymethyl methacrylate-styrene copolymer (P(MMA-co-S)) was synthesized. (P(MMA-co-S)) decomposes to form two free radicals: the stable SG1 nitroxide radical and the copolymer chain end radical. The free radicals at the end of the copolymer chain react with the double bonds on the surface of the VBC-SNP to graft the copolymer onto the nanoparticles. Finally, the free radicals are deactivated by nitrogen oxides to terminate (Fig. 2d) [70].

RAFT can modify polymers with controllable molecular weight and structure on the surface of nanoparticles under mild conditions and is easy to functionalize. RAFT is similar to conventional free radical polymerization except that it requires a RAFT agent. Dusadee Tumnantong et al. used poly (styrenesulfonate-sodium)-RAFT agent to prepare polystyrene-silica nanoparticles (PS-co-RAFT-SiO_2_) through RAFT. Studies have shown that surface grafting improves the thermal and mechanical properties of nanoparticles (Fig. 2e) [71]. Xing et al. first prepared mercaptopropyl modified silica, and then copolymerized 1-vinyl imidazole and acrylic acid by RAFT to form a polymer brush on the surface of silica, which has good heavy metal ion adsorption performance [72].

Grafting has been widely used in the surface modification of nanoparticles. It can not only modify the surface of nanoparticles by selecting different polymers to obtain different properties without affecting the properties of the nanoparticles themselves, but also ensure the controllable introduction of graft chains with high density and precise positioning on the NP surface. In addition, the covalent modification by grafting to the surface of nanoparticles has long-term stability.

**Table 2:** Summary and comparison of surface modification methods

Strategy		Materials	Modified ligand	Effect	Advantage	Disadvantage	Refs.
Adsorption		CaCO_3_	CTAB and sodium oleate	Affect the surface potential of NPs and improve dispersion	simple	Non-covalent bond connection, unstable under external stimuli	[50]
Coupling		TiO_2_	KH550	Improve the dispersion of NPs and increase biocompatibi-lity	Improve the compatibility of inorganic NPs with organic substances, and can be further modified.	The scope of application is narrow, usually used for inorganic NPs with hydroxyl groups on the surface	[61]
		SiO_2_	TESPSA	Improve the dispersion of NPs and enhance surface activity			[55]
Graft							
Light-initiated grafting		SiO_2_	MMA	Improve the stability and surface activity of NPs	Not only can obtain different properties by selecting different polymer modifications, but also have long-term stability.	The operation is complicated and difficult.	[58]
ATRP		SiO_2_	hydrophilic polymer	Improve the dispersion of NPs			[68]
NMP		Starch NPs	P(MMA-co-S)	Improve the dispersion of NPs			[70]
RAFT		SiO_2_	PS	Improve the thermal and mechanical properties of NPs			[71]

In summary, the significance of modifying the surface of nanoparticles by nano-engineering is as follows: (1) Improving the dispersion, stability and biocompatibility of nanoparticles; (2) Improving the surface reactivity of nanoparticles; (3) Changing the physical, chemical and mechanical properties of the surface of nanoparticles. Surface modification can make nanoparticles have more superior properties, become more ideal materials and further customize their functions to have wider applications in different fields.

### Structure design

With the development of nanotechnology, the performance of single material is no longer sufficient to meet complex practical needs, so multifunctional nanomaterials that can integrate multiple functions into a single structure are required. In order to obtain nanomaterials with ideal properties, it is necessary to precisely design the structure and composition of the nanoparticles, and combine the advantages of different materials to achieve the same or better effect as the combination of single materials [73]. At present, several types of nanoparticles have been proposed, including core-shell structure, yolk-shell structure, sandwich structure, satellite core structure, etc., all of which have excellent properties, and it has a wide range of applications in various fields such as catalysis, biomedicine, and energy storage.

The core-shell structure is to coat one or more layers of other materials on the surface of the original nanoparticles. The original nanoparticle is called the core, and the surrounding layer is called the shell. The preparation of the core-shell structure usually uses sol-gel or chemical precipitation to form nucleation sites on the surface of the core for nucleation and growth to form a shell [74, 75]. The shell can change the properties of the original nanoparticles, and improve the stability and dispersibility. In addition, the core-shell structure enables core nanoparticles to possess new properties such as light, magnetism, and electron transfer [76, 77]. Therefore, through the direct combination or synergy of the inner core and the outer shell, the nanoparticle can have a novel combination function or optimize the original performance. In addition, the core-shell structure can easily remove the core, thereby constructing a hollow structure with large storage capacity and low density.

The most important advantage of the core-shell structure is that the shell and the core can interact, so that the core-shell nanoparticles have better performance than single nanoparticles. Li et al. proved that the PPy shell reduces the magnetic coupling effect between ZnFe_2_O_4_ nanoparticles, increases the surface anisotropy, and makes the dielectric constant and permeability better match. Compared with ZnFe_2_O_4_, ZnFe_2_O_4_/PPy core-shell nanoparticles have stronger microwave absorption performance and wider absorption band [78]. Similarly, Christopher J. Serpell found that due to the interaction of electrons and lattices in core-shell nanoparticles, the catalytic performance of bimetallic Au@Pd core-shell nanoparticles is better than the same alloy nanoparticles or a mixture of same metal nanoparticles [79].

The shell based on the core-shell structure changes and protects the surface properties of the original nanoparticles, which is of great significance in the field of drug delivery. At present, targeted drug delivery is one of the research hotspots. The design of targeted drug delivery platforms is mainly to encapsulate drugs on the surface or inside of nanoparticles, and then coat them. The coating can be a simple polymer, such as hydrophilic polyethylene glycol (PEG), to enhance the hydrophilicity of the original nanoparticle surface, prolong the half-life of blood circulation, and make it more effective to target the lesion. The coating can also degrade in response to certain stimuli to release drugs, such as external stimuli such as light and heat, or target cell microenvironment stimuli such as PH. This part will be described in detail later in the targeting part.

**Fig. 3:**
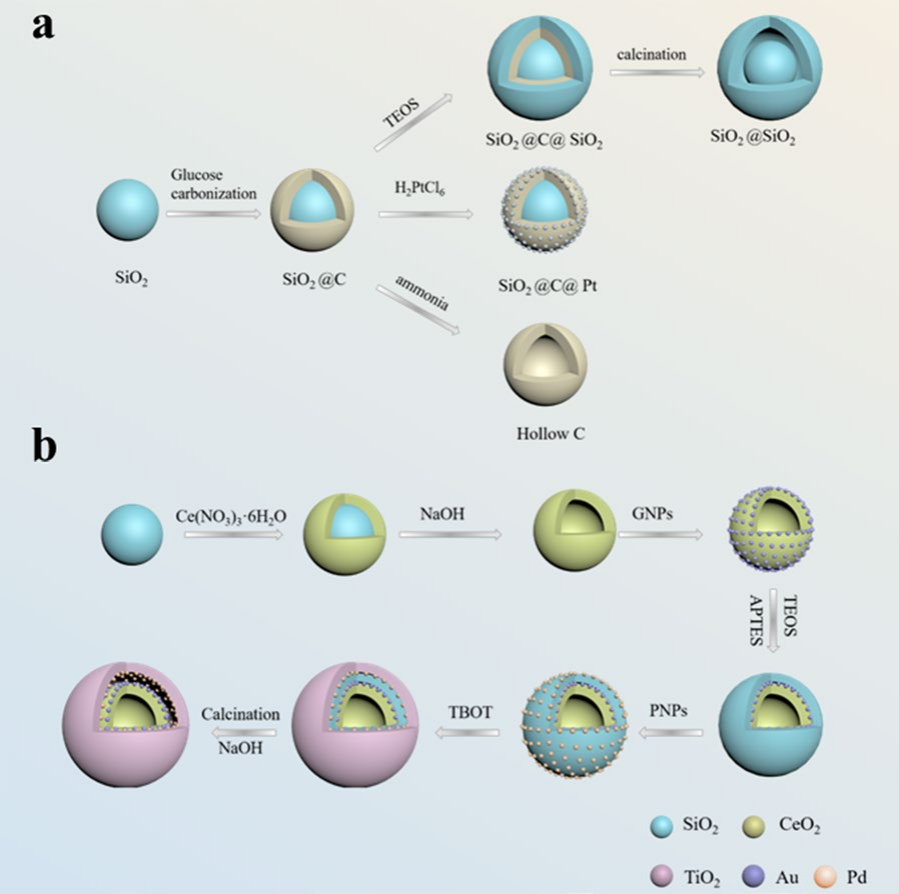
Schematic illustration of the core-shell structure is used as a template to prepare **a** yolk-shell, hollow and satellite-core structures; **b** multi-shell hollow nanostructure

Core-shell structured nanoparticles can also be used as templates to design and prepare nanoparticles with other types of structures. Wan et al. prepared SiO_2_@C nanoparticles by a hydrothermal carbonization method, and then obtained yolk-shell structure, hollow structure or satellite core structure through different subsequent preparations. They used TEOS to form another layer of silica shell on the surface of SiO_2_@C particles, calcined at high temperature to remove the carbon shell, and obtained SiO_2_@SiO_2_ spheres with a void in the two SiO_2_ shells, that is, the yolk–shell structure. Use ammonia water to dissolve the silica core to form a hollow carbon capsule. In addition, they used the carbon shell with strong reducing ability on the surface of SiO_2_@C to reduce the noble metal salt solution, so that the noble metal nanoparticles were loaded on the SiO_2_@C carbon shell in situ, and the nanoparticles with the satellite-core structure were obtained (Fig. 3a) [80]. Liu et al. designed a multi-shell hollow nanostructure based on the core-shell structure, which has a large specific surface area and high load capacity. Firstly, SiO_2_@CeO_2_ microspheres were prepared by the polyol-assisted hydrothermal method, and then deposited layer by layer, followed by alkali etching to obtain the double shell layer @CeO_2_/Au@Pd/TiO_2_ nanospheres (Fig. 3b). The double shell has different components, and different noble metal nanoparticles are loaded on the outer surface of the metal oxide inner shell and the inner surface of the outer shell. The multiple interactions and synergistic effects between the noble metal nanoparticles and the metal oxide shell can effectively improve the catalytic activity, and because the noble metal nanoparticles are attached to the metal oxide shell, they are not easy to aggregate with each other, which improves their catalytic stability [81].

The yolk-shell structure is composed of a shell and a movable core. There is a cavity between the shell and the core, which can be described as core@void@shell structure. The synthesis method of yolk-shell structure can be roughly classified into hard template method, soft template method and self-template. The hard template method is the most commonly used method. The surface of the core nanoparticles is coated with template before continuing to cover the shell, and then the template is removed. The soft template method uses vesicles self-assembled by amphiphilic molecules. Compared with the hard template, the template does not need to be removed [82, 83]. The yolk-shell structure has the common advantages of hollow structure and core-shell structure, with large surface area, large void space and high load capacity. In addition, (1) The shell of the yolk-shell structure allows guest molecules to diffuse in and interact with the movable core; (2) The movable core usually has a large number of active sites to ensure the efficiency of the reaction; (3) The shell can play a protective role and inhibit irreversible aggregation between the cores; (4) The hollow part between the shell and the core can provide space for storage or reaction [76, 82, 84–86]. Therefore, compared with traditional core-shell nanostructures, the yolk-shell structure has better performance.

As we all know, noble metal nanoparticles have excellent catalytic performance as a catalyst, but because they are very easy to aggregate, the catalytic performance will be greatly reduced. The yolk-shell structure can effectively solve this problem. The noble metal nanoparticles can be loaded on the original core, or the noble metal nanoparticles themselves can be used as the core and encapsulated in the shell. The reactant can diffuse into the internal cavity through the shell and be catalyzed by the noble metal nanoparticles, and the original core or shell protects the noble metal nanoparticles, prevents their mutual aggregation, improves their stability, and improves their catalytic activity. Wang et al. prepared Au@ mesopore SnO_2_ yolk-shell NPs. Au NPs move freely in the SnO_2_ shell, and there are abundant pores between SnO_2_ NPs. The reactant molecules can effectively contact with Au NPs for catalysis through the abundant pores of the SnO_2_ shell, and the SnO_2_ shell can separate the Au NPs from each other well, avoiding the aggregation of Au NPs, thereby improving the stability and catalytic activity of the catalyst [85]. Du et al. designed a yolk-shell structure with dendritic porous core and mesoporous shell. Many noble metal NPs are loaded on dendritic porous silica spheres (DPSSs), and the outer protective mesoporous silica shell (MSS) has vertically arranged pores. On the one hand, the dendritic structure of the core provides a large specific surface area for loading ultrafine Au or Pt NPs, and a large number of loaded noble metal nanoparticles ensure the effectiveness of the catalytic reaction. On the other hand, the pore channel of the core also acts as a physical barrier to separate and restrict the loaded noble metal nanoparticles, avoid their aggregation, and improve the stability of the catalyst. In addition, MSS with vertically arranged pores can shorten the diffusion path of reactants, which is beneficial to the occurrence of catalytic reactions. The research results show that the yolk-shell structure effectively improves the activity and stability of the catalyst [87].

The yolk-shell structure material can also be used for lithium-ion batteries. The large surface area and large void space of the yolk-shell structure can provide more active sites for lithium-ion storage and increase battery capacity. More importantly, the hollow area can buffer the volume change caused by the reaction process of the lithium-ion battery, improve the structural stability, and give it better cycle performance. Leng et al. designed a multi-layer porous shell yolk-shell structure. Both the multilayer shell and the core can be in contact with the electrolyte, providing more active sites for lithium-ion storage, so the reversible capacity is significantly improved. Secondly, the porous structure of the shell can provide a penetration path for the electrolyte and shorten the lithium-ion diffusion path, so it has excellent rate performance. In addition, the voids inside the yolk-shell structure serve as a buffer for volume changes, increasing the cycle life [88]. Therefore, the yolk-shell structure material they designed has excellent electrochemical properties such as high reversible capacity, long cycle life, and good rate performance.

**Fig. 4:**
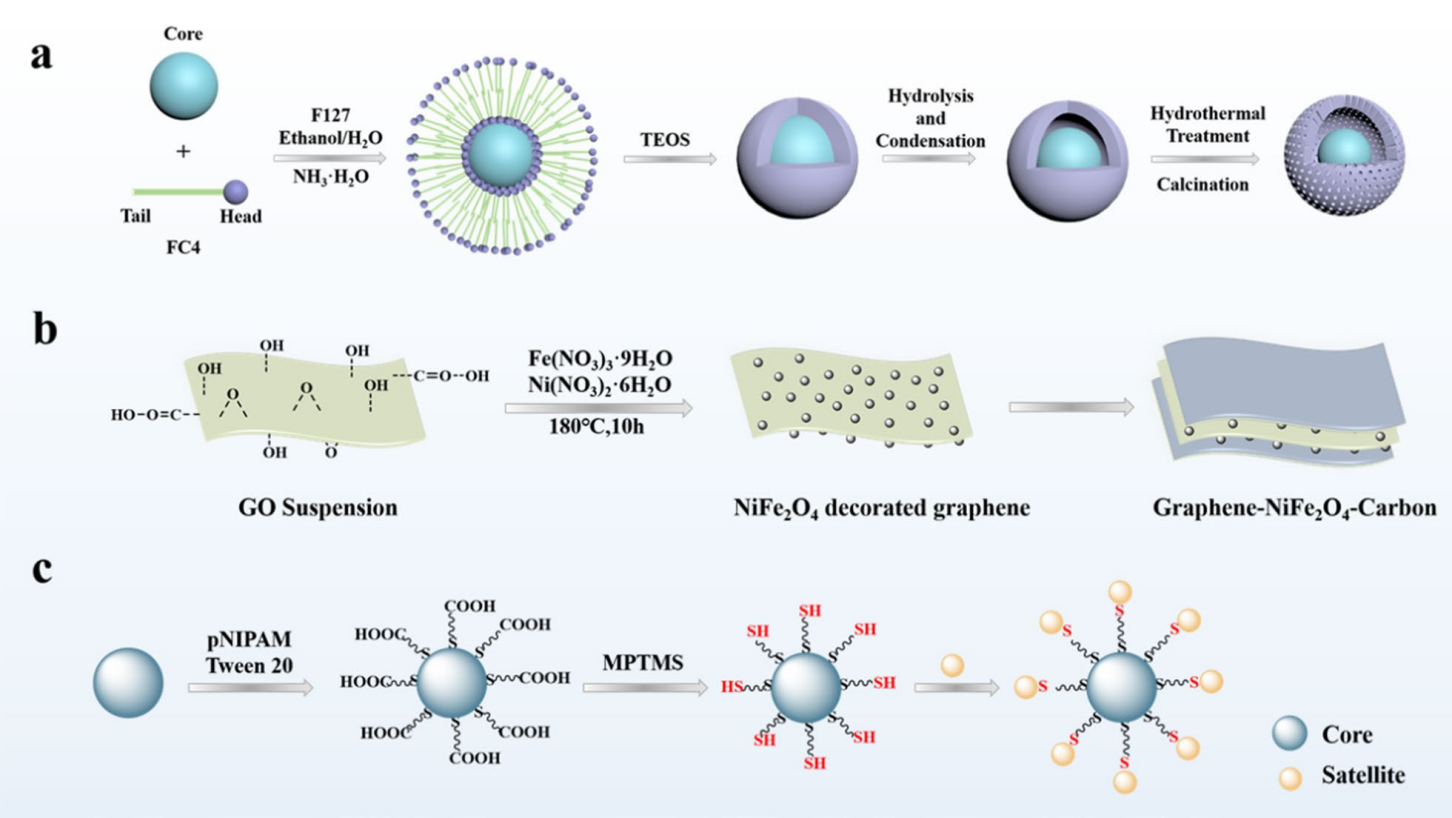
**a** Schematic illustration of the preparation of yolk shell structures with mesoporous shells. **b** Illustration of the synthesis procedure for graphene-NiFe_2_O_4_-carbon nanocomposites with a sandwich structure. **c** Illustration of the synthesis procedure for thermo-responsive plasmonic core-satellite nanostructures

The cavity and porous shell of the yolk-shell structure make it an attractive carrier for drug delivery. In addition, due to its core@void@shell structure, it can be used for graded drug delivery to achieve safe and effective drug delivery. The initial rapid release of part of the drug is used to alleviate the condition, and then the remaining dose is subsequently released to maintain or optimize the therapeutic effect. In addition, different drugs can be loaded in different areas for sequential delivery. Liu et al. encapsulated different nanoparticles in porous silica shells to obtain yolk-shell structured nanoparticles, such as silica balls, mesoporous silica NPs, gold particles, magnetic particles, etc. (Fig. 4a). The obtained yolk-shell nanoparticles can release the loaded drugs in three steps. First, the drug adsorbed on the shell surface is quickly released, reaching a plateau, and then the drug in the inner hollow space is released again, reaching another plateau, and finally the drug adsorbed on the core is released [86]. The yolk-shell structure can achieve precise controlled release of drugs.

The sandwich structure is a multifunctional composite structure constructed by attaching thin rigid panels on both sides of low-density materials. It has the advantages of low density, large specific surface area, and high structural stability [89]. In addition, the sandwich structure can effectively increase the rigidity of the structure without adding weight, and can improve the energy absorption and storage properties of the material [90]. These advantages of the sandwich structure make it widely used in lithium-ion batteries, biomedicine and other fields.

The excellent performance of the sandwich structure depends on the configuration of the structure and the choice of the sandwich material. By precisely controlling the structural configuration and adjusting the inner core material and the panel material, the material has high specific surface area while having high stability, so that there are more active sites for reaction, better performance and longer life. Lv et al. designed a graphene nanosheet/NiO (GNS/NiO) with a multilayer sandwich structure. GNSs serve as a substrate to fix NPs and prevent NPs from re-aggregating, while NPs serve as spacers to prevent GNSs from re-stacking to form a stable structure, effectively maintaining the active surface, leaving a stable and open channel for ion transfer, and the sandwich structure provides a buffer for volume changes. Therefore, this nanomaterial can be effectively applied to electrochemical energy storage [91]. Elham Kamali Heidari and his colleagues grow NiFe_2_O_4_ nanoparticles on both sides of graphene sheets and then apply carbon coatings (Fig. 4b). The graphene layer not only improves conductivity, but also acts as a buffer to relieve the stress caused by volume expansion. In addition, the NiFe_2_O_4_ nanoparticles grown on the graphene layer can maintain good dispersion, avoid aggregation, and have a larger specific surface area to provide more active sites for lithium-ion reactions. The carbon coating is used to prevent NiFe_2_O_4_ nanoparticles from falling off the graphene matrix and improve the cycling stability of the electrode. The carbon layer can also be used as a stress buffer and an electronic conductor to further improve the performance of the electrode. Based on the synergistic effect of each layer of sandwich structure nanoparticles, a lithium-ion battery negative electrode material with significantly enhanced cycle stability and electrochemical performance is obtained [92]. Guo et al. combined Fe_3_O_4_-COOH nanoparticles with polyallylamine hydrochloride (PAH) and inserted them into graphene oxide-COOH sheets through electrostatic interaction. The obtained multilayer sandwich structure hinders the accumulation of GO-COOH flakes, increases the specific surface area, increases the adsorption active sites, and improves the adsorption capacity. In addition, the structure has good stability and can be used repeatedly [93].

The core-satellite structure is to grow many smaller nanoparticles on the surface of larger nanoparticles. The smaller nanoparticles are called satellites, and the larger nanoparticles serve as the core. The core-satellite structure is prepared by forming heterogeneous nanoparticles on the surface of the core particles by chemical deposition, seed growth, chemical reduction, etc. By controlling the surface and free energy of the material, the growth position can be controlled [94]. Core-satellite structured nanoparticles usually have excellent optical properties and have important applications in biosensing, imaging and other optical fields.

Local surface plasmon resonance (LSPR) is the collective oscillation of free electrons on the surface of nano-scale metals or particles under the same light wave as their resonance frequency. When a substance is on the surface of a nano-scale metal structure/particle or is very close to the surface, the Raman scattering signal of the substance will be significantly enhanced. This phenomenon is called surface-enhanced Raman scattering (SERS). The enhancement of SERS is the amplification caused by the enhancement of the local electromagnetic field generated by LSPR, and the enhancement factor can reach 10^14^, realizing single-molecule detection. In the nanoparticles designed by Xiong et al., AuNPs are randomly distributed on the surface of the CGNR core as satellites, and they have strong surface plasmon coupling. By increasing the size and number density of satellite nanoparticles or reducing the thickness of the silica spacer between the nucleus and the satellite particles, the plasma band can be redshifted and the surface enhanced Raman scattering can be enhanced, which is effectively used in sensing and imaging [95].

The core and satellite of the core-satellite structure can be used as donors and acceptors, respectively, to effectively transmit electrons and energy for biosensing. Resonance energy transfer (FRET) is the transfer of energy from the donor’s excited state to the acceptor through dipole-dipole interaction without radiation resonance. The energy transfer efficiency is extremely sensitive to distance, and a highly sensitive nanometer can be made. Han et al. designed a temperature-responsive polymer-connected core-satellite structure (Fig. 4c). As the temperature rises, the polymer chain shrinks and the distance between the core and the satellite decreases, which causes the surface plasmon coupling band to redshift and increase. Therefore, this temperature-responsive polymer-connected core-satellite structure can be used as a temperature-responsive biosensor [96]. Temperature-responsive polymers can be replaced with other stimulus-responsive polymers that are sensitive to pH, light, or redox, for broader biosensing.

The core-satellite structure of nanoparticles can also compound multiple functions, integrating the excellent performance of each building block into the nanoparticles. It can be used for multi-modal imaging guided therapy and applied in the field of biomedicine. Ma et al. designed nanoparticles with a hollow-satellite shell structure to integrate multiple functions into one. The MRI contrast agent Fe_3_O_4_ is used as a satellite to be distributed on the organic silicon nanocapsule (OSNC) shell for imaging. The OSNC shell provides good dispersion and biocompatibility in water, and the hollow part can be used to encapsulate the therapeutic agent. The nanoparticles they designed can effectively treat cancer through high-intensity focused ultrasound therapy guided by magnetic resonance imaging [97].

## Nano-engineering in tumor treatment

Nano-engineering can customize the function of nanoparticles, endow them with various desired properties, and make them have broader application prospects. In this section, we will focus on the application of nano-engineering in tumor treatment. Targeted delivery of therapeutic agents, tumor drug resistance and immunosuppression are the main difficulties and treatment obstacles for the application of nanoparticles in tumor therapy, and nano-engineering nanomedicines with customized functions can solve these problems well to obtain the best tumor treatment effect.

### Targeted delivery

Because traditional delivery methods have low selectivity for tumor cells, high metabolic rate, low treatment efficiency, and high toxicity and side effects on normal tissues [17], nanomedicines have entered people’s field of vision. Nanomedicines has a large specific surface area, adjustable morphology and surface properties, can be used for targeting and controlled/sustained release, and can protect the payload from degradation, improve the stability and half-life of the payload in the blood circulation, and at the same time, it can concentrate multiple functions into one delivery system, which plays an indispensable and important role in tumor therapy [6, 98, 99]. However, most of the nanomedicines will be phagocytosed and cleared by the reticuloendothelial system (RES) in the blood circulation, and have no specific recognition ability for tumors, which will reduce the accumulation of nanoparticles at the tumor site and damage the RES organ [6]. Nano-engineering nanomedicines can overcome the above-mentioned problems, and maximize the effective delivery of therapeutic agents to the tumor area. This is mainly based on two types of targeting: passive targeting and active targeting.

#### Passive targeting

Passive tumor targeting is generally achieved through the enhanced permeability and retention (EPR) effect. The EPR effect is a phenomenon in which the accumulation of nanoparticles of suitable size in tumor tissues is much higher than that in normal tissues [6]. The reason for this phenomenon: In the process of tumor tissue growth, a large number of tumor blood vessels are needed to provide oxygen and nutrients. However, due to the rapid growth, the tumor blood vessels have not matured and differentiated, and there are gaps between the blood vessel walls. Nanoparticles can penetrate into the tumor tissue through the gap. At the same time, due to the imperfect development of the return system such as lymphatic vessels, it is difficult for the nanoparticles to exit the tumor tissue, and the nanoparticles can accumulate in the tumor tissue and then play a role. Matsumura and Maeda and their colleagues first discovered the EPR effect [100], and they have been playing an extremely important role in cancer treatment since then. The EPR effect is affected by the size, shape, and surface properties of nanoparticles [101, 102]. Through nano-engineering, controlling the size of nanomedicines, or modifying their surface, such as hydrophilic groups and biomimetic preparations, which can largely avoid the phagocytosis and removal of nanomedicines by RES, and prolong the time in the blood circulation, so that it has more opportunities to gather in the tumor area through the EPR effect, so as to exert its best effect.

In order to enhance the accumulation of nanomedicines at the tumor site through the EPR effect, the most commonly used method is to change the size of the nanoparticles. When the size of nanoparticles is too small (< 4-5 nm), they will be filtered by the kidneys and excreted from the body. When the size of nanoparticles is too large (> 200 nm), they are easy to be recognized by RES, and it is difficult to penetrate the tumor tissue through the gap of the blood vessel wall. Therefore, the size of the nanomedicines should be between 10 and 200 nm, which have a long half-life in blood circulation and effectively enter the tumor tissue through the EPR effect [6, 99]. Liu et al. prepared AuNC@CBSA@HA nanoparticles (100 nm, 200 nm, 300 nm) of different sizes by adjusting the ratio of ligands. They proved that AuNC@CBSA@HA with a size of 100 and 200 nm has similar passive tumor accumulation, which is about 1.5 times of the accumulation of 300 nm [103]. Hye Lan Kim prepared monodisperse silica nanospheres of different sizes and studied the effect of size on the accumulation of nanoparticles in mouse U87MG tumors. Their research showed that the accumulation of MSN with a size of 100–150 nm in the tumor site is 4-6.5 times higher than that of MSN with a size of < 30 nm or > 300 nm [104]. By controlling the size of nanoparticles, the EPR effect can be enhanced.

The above-mentioned researches all directly adjust the size of nanoparticles to enhance the EPR effect. However, studies have shown that nanoparticles with a larger size (> 100 nm) have a longer blood circulation time and can remain in the blood circulation for a long time, so that the nanoparticles can effectively accumulate in the tumor tissue. Nanoparticles with a smaller size (< 50 nm) have strong permeability to tumor tissue and can quickly spread to the entire tumor tissue [105]. Nanoparticles of a single size cannot meet the two requirements of long circulation and high permeability at the same time. Therefore, nanomedicines with switchable sizes have attracted people’s attention. This method is usually associated with active targeting, so we will discuss it in detail later in the active targeting section.

After nanomedicines enter the blood circulation, they will interact with blood components and be phagocytosed and removed by RES. This problem can be effectively avoided by modifying the surface of nanomedicines. The most commonly used strategy is to modify the nanomedicines with hydrophilic PEG, which increases the hydrophilicity of the surface of the nanomedicines while reducing the ability to bind to proteins in the blood, making the nanomedicines “invisible”, thereby reducing the uptake of RES, prolonging the circulation of nanomedicines in the blood vessel, and targeting the tumor through the EPR effect.

Wang et al. used PEG derivatives to modify nanoparticles. Compared with unmodified nanoparticles, after modification with PEG derivatives, the binding rate of nanoparticles and proteins is reduced by more than half, prolonging their residence time in the blood circulation, and improving the stability of drug-loaded nanoparticles [106]. However, only PEG modification is not enough to increase the blood circulation half-life of nanomedicines. Therefore, based on PEG, a more effective strategy has been developed. Gao et al. used hydrophilic PEG and hydrophobic Poly(2-Vinylpyridine) (P2VP) fragments to prepare a series of microphase-separated mixed-shell micelles (MSMs) with different hydrophilic-hydrophobic ratios. Among them, MSMs-50 with a hydrophilic/hydrophobic ratio of 5/5 has a much higher blood retention time than other MSMs. Their research showed that by simply adjusting the hydrophilicity and hydrophobicity of nano-engineering, long-cycle nanoparticles can be obtained [107]. Amphiphilic materials have both positively and negatively charged groups, but the overall display is neutral, which can effectively replace PEG, resist non-specific protein adsorption, and avoid interaction with blood components [6]. Ou et al. used amphiphilic materials poly(2-methacryloyloxyethyl phosphorylcholine) (PMPC) combined with poly(b-amino ester) (PAE) to form MSMs on the surface of nanoparticles. Under normal blood pH (7.4), PAE chain invades, and the amphiphilic material prolongs the blood circulation half-life of nanoparticles. In the slightly acidic tumor site, PAE is protonated and the surface is positively charged, which enhances cellular uptake [108].

**Fig. 5:**
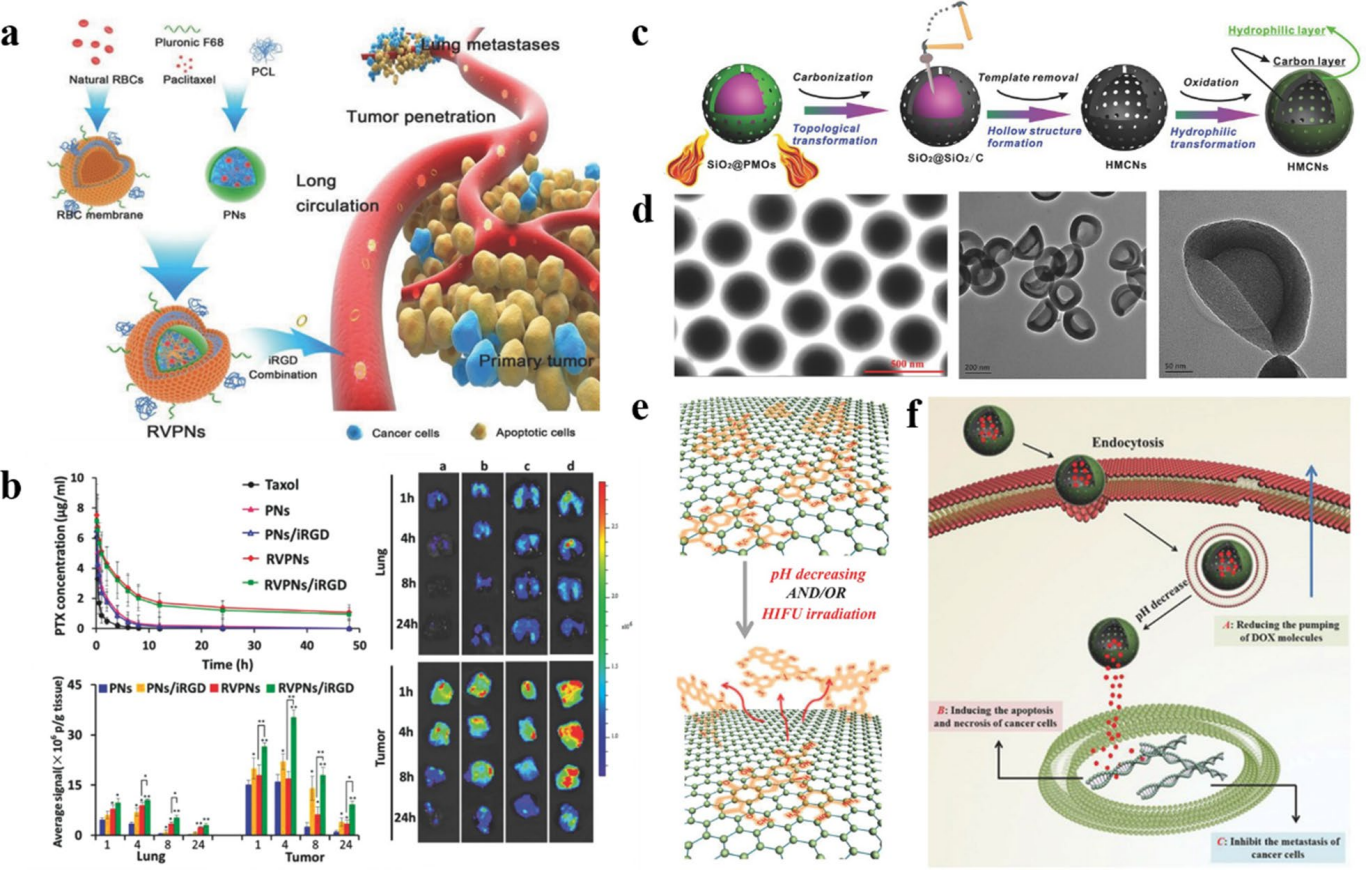
**a** Schematic illustration of RBC-mimicking NPs with extended blood circulation and enhanced tumor penetration. **b** Pharmacokinetics and biodistribution of RBC-mimicking NPs in 4T1 tumor-bearing mice. Reproduced with permission [111]. Copyright 2016, WILEY-VCH. **c** Schematic illustrations of the synthetic procedures for HMCNs. **d** TEM images of SiO2@SiO2/C and HMCNs. **e** Schematic illustration of HMCNs interacting with DOX via π-stacking, and DOX release from HMCNs. **f** Schematic illustration of DOX-loaded HMCNs exerting anti-tumor efficacy. Reproduced with permission [112]. Copyright 2014, WILEY-VCH.

Another common strategy is to use cell membranes to coat nanomedicines [109]. Red blood cell (RBC) membrane has a “self-labeled” transmembrane protein CD47 [110], through the coating of the red blood cell membrane (RBCm), the nanomedicines can be disguised as cells, making them invisible in the blood circulation, avoiding the uptake of phagocytes, and achieving long circulation. Su et al. prepared nanoparticles that mimic red blood cells (RVPNs), using RBC mimic vesicles (RV) to coat hybrid polymeric nanoparticles (PNs) loaded with the anticancer drug paclitaxel (PTX). In vivo pharmacokinetic studies have shown that compared with PNs, RVPNs have increased blood circulation half-life several times. In their research, RVPNs can also effectively penetrate the tumor area by combining with the tumor penetrating peptide iRGD (Fig. 5a) [111]. The above research directly uses RBCm to coat nanomedicines, uses the proteins and sugars on the RBCm to avoid recognition by the immune system, and the use of RBC morphology can also prolong blood circulation time. Chen et al. controlled synthesis of mesoporous carbon nanoparticles (MCNs) with RBC morphology and hollow core/mesoporous shell nanostructures. Through TEM characterization, it can be known that the mesoporous carbon shell forms a unique red blood cell surface shape (Fig. 5c). Nanoparticles with RBC morphology can achieve long blood circulation time and have more opportunities to be delivered to the tumor site through the EPR effect [112]. Nishit Doshi et al. also studied the synthesis of materials that mimic RBC, including the synthesis of particles with RBC biconcave disc-like morphology through templates, or the synthesis of particles with RBC intrinsic hemoglobin through layer-by-layer assembly (LBL).These materials are good candidates for targeted delivery [113].

In conclusion, by adjusting the size and surface properties of nanomedicines, it can effectively improve the stability of the material, avoid the phagocytosis and clearance of nanoparticles by RES, prolong the half-life of blood circulation, and make the nanomedicines have more opportunities to target and aggregate to the tumor area through the EPR effect.

#### Active targeting

Nanomedicines can be passively targeted and delivered to tumor tissues through the EPR effect, but as mentioned above, the EPR effect depends on the characteristics of tumor blood vessels. In tumor areas where blood vessels have not yet formed, or large tumor central areas or necrotic areas where blood vessels are insufficient, nanoparticles cannot be effectively reached by the EPR effect [114], and only through passive targeting, most nanoparticles still cannot effectively aggregate to the tumor area. Therefore, when passive targeting is insufficient for effective delivery to the tumor area, active targeting enters people’s field of vision.

There are many studies on active targeting, which can be roughly divided into various receptor-mediated targeting and stimulus-response targeting strategies [8, 115, 116]. Nano-engineering nanomedicines designed according to the above targeting strategies play an important role in tumor treatment. Special emphasis was placed on stimulus-response-targeted nanodrugs as a new active targeting strategy, and their " smart " response change characteristics make it have broad research potential.

Receptor-mediated targeting is to specifically recognize receptors overexpressed on the surface of tumor cells by modifying targeted ligands on the surface of nanoparticles, thereby increasing the accumulation of nanoparticles at the tumor site [117]. The main receptors on the surface of tumor cells include folate receptor (FR) [118, 119], transferrin receptor (TfR) [120], epidermal growth factor receptor (EGFR) [121], human epidermal growth factor receptor 2 (HER2) [122], Cluster of differentiation (CD) receptors-CD44 [123], αvβ3 integrin receptor [124, 125], estrogen receptor (ER), biotin receptor, interleukin (IL) receptor, etc [126]. By modifying the ligands corresponding to the above receptors on the surface of nanomedicines, tumor cells can be specifically targeted and the accumulation of nanomedicines can be promoted, thereby improving the effect of tumor treatment.

Stimulus response targeting is subtle changes in nanoparticles under internal (tumor microenvironment) and external stimuli (magnetic field, light, etc.), thereby effectively accumulating and functioning at the tumor site [8, 127]. Stimulus-responsive targeted nanodrugs can often be associated with “smart switches” and are currently a research hotspot. Therefore, this section focuses on the application of nano-engineering nanomedicines in the field of stimulus-response targeting.

The tumor area has a unique microenvironment, including overexpression of certain enzymes, high levels of glutathione (GSH) in the cell, and low pH in the tumor area [6, 98]. Based on these characteristics, stimulus-responsive nanomedicines that specifically respond to the tumor microenvironment can be designed and delivered in a specific location. Such stimulus-responsive nanomedicines are of great significance to effectively accumulate in the tumor area and reduce toxicity and side effects on normal tissues. Therefore, based on nano-engineering, nanomedicines can be prepared by using ligands that respond to the characteristics of the tumor area, so as to achieve the purpose of directional delivery to the target area.

**Fig. 6:**
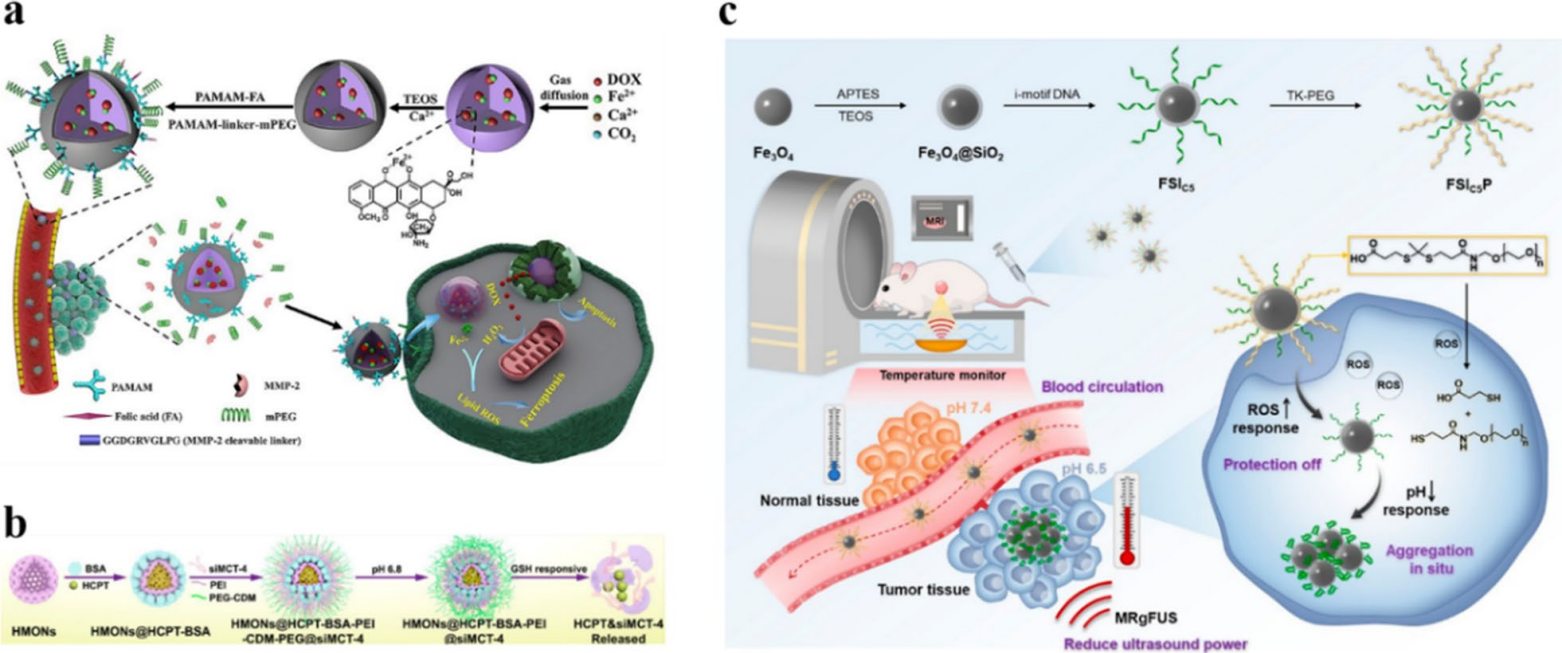
**a** Schematic illustration of the synthesis of ACC@DOX.Fe^2+^-CaSi-PAMAM-FA/mPEG and its therapeutic effect. Reproduced with permission [129]. Copyright 2020, American Association for the Advancement of Science. **b** Schematic illustration of the synthesis of NPs and GSH-responsive degradation. Reproduced with permission [134]. Copyright 2020, American Chemical Society. **c** Schematic diagram of the synthetic route of FSIC5P and the function of FSIC5P in response to ROS and pH in tumor tissues. Reproduced with permission [139]. Copyright 2022, American Chemical Society

Enzymes play an indispensable role in life activities. The expression of enzymes is different in different tissues of different states. In the tumor area, the expression of matrix metalloproteinase 2 (MMP-2) is much higher than that of other normal tissues [128]. This feature has been widely used in the design of targeting structures. Nanomedicines obtained by surface modification or structural design with MMP-2 sensitive peptides can utilize the mutual recognition between enzymes and substrates to construct a highly sensitive targeting system to target tumor cells with high MMP-2 expression. Xue and his colleagues used MMP-2 sensitive peptides to modify the multifunctional dendrimer containing PEG on the surface of the delivery nanoplatform containing folic acid (FA) (Fig. 6a). PEG prolongs the blood circulation time of the delivery system. The MMP-2 sensitive peptide breaks in response to the high expression of MMP-2 in the tumor area, thereby removing the PEG shield, and then effectively targeting the tumor through FA, accumulating in the tumor and exerting therapeutic efficacy [129]. Enzyme-responsive degradation of nanomedicines is also available based on MMP-2, and this part can be linked to the size-switchable nanomedicines mentioned above. The smaller size nanoparticles are combined with itself or other substances through the MMP-2 sensitive peptide to form larger size nanoparticles, and the larger size is used for long circulation. In the tumor area, the MMP-2 sensitive peptide specifically binds to the highly expressed MMP-2 and breaks, allowing smaller-sized nanoparticles to be released for effective penetration. For example, He et al. used MMP-2 sensitive peptide (pp) to link MSN to star-shaped PEG to construct a nanoparticle with a special structure. The longer blood circulation half-life of MSNs-pp-PEG can effectively accumulate nanoparticles in the tumor area. After reaching the tumor area, pp is destroyed in response to MMP-2, MSNs nanoparticles are released, and the smaller size of MSN quickly spreads throughout the tumor tissue. The penetration of fluorescently-labeled nanoparticles in the 3D model shows that MSN has the highest penetration rate compared to the larger-sized MSNs-PEG. The final accumulation of MSNs-pp-PEG in tumors was 1.74 times and 2.05 times higher than that of MSNs and MSNs-PEG, respectively [130]. Therefore, the accumulation of the size-switchable nanomedicines in tumor is significantly increased, and it can be expected that it will have the best therapeutic effect.

GSH is a tripeptide composed of glutamic acid, cysteine and glycine, containing γ-amide bond and sulfhydryl group, which is overexpressed in tumors. And it has two forms, oxidized state (G-S-S-G) and reduced state (GSH), which can be used to construct redox-sensitive targeting systems and have important applications in various tumor-targeted nanomedicines [131, 132]. Nanomedicines with disulfide bonds can be degraded in response to GSH, so by designing the structure, a GSH-triggered targeted delivery system with disulfide bonds can be constructed [133]. Li et al. used disulfide bonds to connect the silica network to obtain mesoporous silicone nanoparticles (MONs), and modified PEG on the surface while loading anticancer drugs to construct a targeted drug delivery system (Fig. 6b). The drug delivery system has a long blood circulation half-life, and the disulfide bonds in MONs can be broken in response to the over-expressed GSH in tumor cells to release drugs for targeted delivery [134]. Chen et al. designed cationic block copolymers with PEG and disulfide bonds to load genetic biological macromolecules through electrostatic interactions. In the microenvironment with high levels of GSH in the tumor area, after the disulfide bond is cleaved in response to GSH, the cationic polymer is transformed into a neutral polymer, and the electrostatic interaction disappears, so that genetic biological macromolecules can be effectively released [135]. In addition, GSH consumption can reduce the high redox state of the tumor site and effectively improve the tumor treatment effect [136, 137].

Low pH is one of the important characteristics of tumor areas. Due to the rapid growth of tumor cells and insufficient oxygen supply, the anaerobic respiration of the cells causes the accumulation of lactic acid, which makes the pH of the tumor microenvironment significantly lower than the surrounding normal tissues. Therefore, the pH difference between tumor tissue and normal tissue can be exploited to design a pH-responsive delivery system to achieve targeted delivery and efficient accumulation of nanomedicines in the weakly acidic environment of tumors. Xie et al. coated the surface of magnetic nanoparticles with calcium carbonate and loaded photothermal agent to construct pH-sensitive targeted delivery system. The calcium carbonate shell decomposes in response to the acidic microenvironment of the tumor, and the release rate of the photothermal agent increases significantly with the decrease of pH. Based on this pH-responsive delivery system, magnetic nanoparticles and photothermal agents can be efficiently accumulated in tumor tissues, so that effective diagnosis and treatment can be performed [138]. pH targeting is usually combined with other active targeting. pH targeting is often combined with other active targeting. Zhang et al. modified reactive oxygen species (ROS)-responsive methoxy polyethylene glycol (TK-PEG) and pH-responsive DNA cross-linkers on Fe_3_O_4_ nanoparticles to prolong blood circulation and improve tumor accumulation and retention of nanoparticles. In the tumor area, the labile TK-PEG layer was shed off in response to overexpressed ROS, after which DNA folded in response to tumor acidity and drove nanoparticles’ aggregation, accumulating at the tumor site, thereby enhancing magnetic resonance imaging and ultrasound ablation (Fig. 6c) [139]. Pharmacy Department and others also designed a pH and GSH dual response nanomedicines through surface modification. In acidic and redox environments, nanomedicines can respond to pH and GSH to release doxorubicin (DOX). Compared with free DOX, its cytotoxicity to MCF-7 cells is 2.14 times higher, while reducing its toxicity to normal tissues [140].

**Fig. 7:**
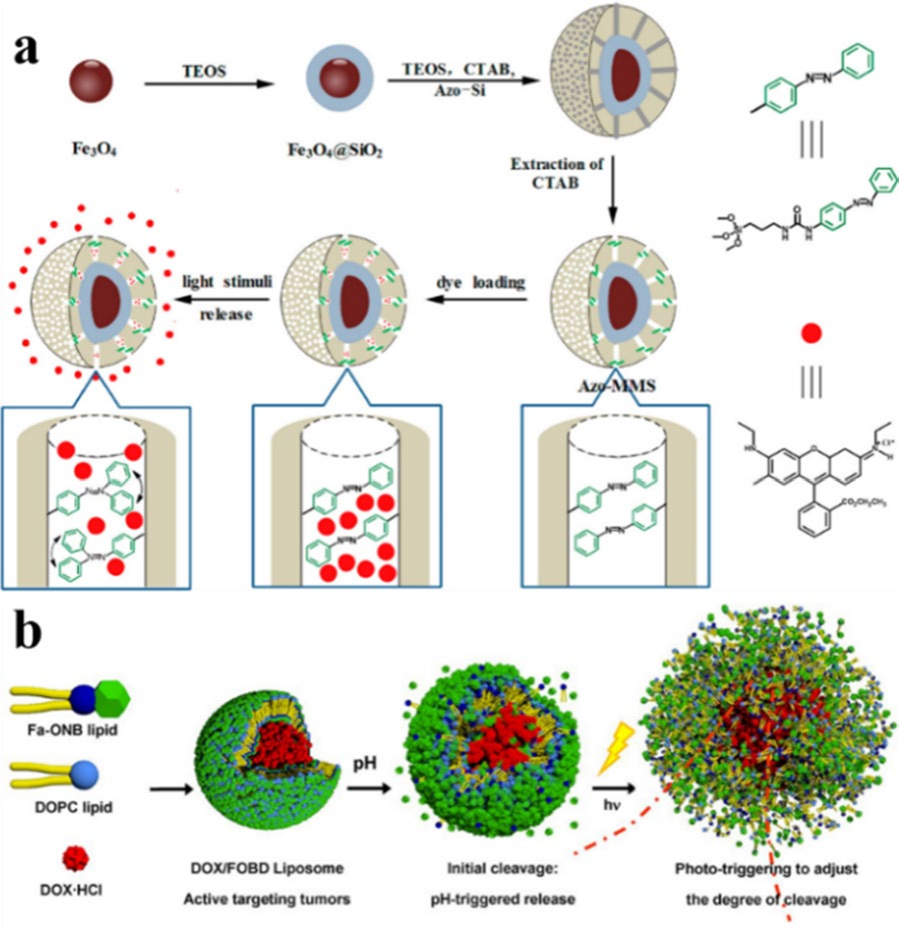
**a** Schematic diagram of the preparation and controlled release process of magnetic and optical targeted delivery systems. Reproduced with permission [142]. Copyright 2012, American Chemical Society. **b** Schematic illustration of the assembly of DOX/FOBD liposomes and liposomes degradation in response to pH and light. Reproduced with permission [143]. Copyright 2019, American Chemical Society

External stimulus targeting is in response to external factors, such as magnetism, light, etc., to make nanomedicines aggregate at specific locations. It has high local accuracy, and can be accurately released by manual adjustment. At present, magnetic nanoparticles (MNPs) for magnetic targeting have attracted a lot of attention. Magnetic targeting usually uses magnetic nanoparticles as a carrier, placing an external magnetic field at a position corresponding to the tumor area in vitro, and directing the magnetic nanoparticles to the tumor site to effectively accumulate. The study of Michael Chorny et al. showed that when MNPs are delivered to arteries under the uniform magnetic field, the accumulation of MNPs can be 4–10 times higher than that of the non-magnetic control group [141]. Wang et al. designed an azo-magnetic mesoporous silica nanoparticle for magnetic and optical dual targeted delivery. On the one hand, the use of magnets to adsorb nanoparticles proved that nanoparticles can be guided to any specific site for targeted aggregation in the presence of an external magnetic field. On the other hand, the covalently modified azobenzene derivatives in the pores of MSN have both cis and trans configurations, and both absorb light at 450 nm. Using the dynamic swing caused by the cis-trans configuration of the azobenzene chain under light stimulation, the substances encapsulated in the mesopores can effectively diffuse out (Fig. 7a) [142]. This light-controlled delivery system with a “switch” can use a specific wavelength of light to turn on the release switch at a specific tumor site, thereby targeted delivery. Some substances that can react under light can also be used to design light-responsive delivery systems. Liu et al. used lipids that are degradable in response to light and pH to prepare micelles as nanocarriers. The o-nitrophenyl ester bond can be broken in response to pH and light to degrade the micelles and release the loaded substances, and by changing the illumination time or wavelength, manual control of precise local release can be achieved. Compared with the non-responsive control group, the dual-response targeting nanocarriers significantly increased the accumulation in tumor tissues (Fig. 7b) [143].

In short, nano-engineering plays an important role in various targeted delivery of nanomedicines. Nano-engineering can endow nanomedicines with better targeting properties through functional customization for efficient delivery to tumor areas. Thus, the tumor treatment effect is improved and the toxicity and side effects to normal tissues are reduced, so that the treatment has higher efficacy and safety.

### Resistance

Chemotherapy is one of the most common and effective ways to treat cancer. However, the efficacy of chemotherapy is limited by the drug resistance of tumor cells. Drug resistance refers to the resistance of tumor cells to chemotherapy drugs, which will significantly reduce the therapeutic effect of chemotherapy drugs. Drug resistance can be divided into primary and acquired. Primary drug resistance refers to the ability of tumors to evade initial treatment and is generally thought to be due to innate genetic alterations, or the rapid adaptation of cells to therapy; Acquired drug resistance refers to the decline of tumor treatment efficacy after long-term treatment, which may be due to genetic mutations in some cells during tumor treatment, resulting in a small number of resistant cells, which survive and proliferate after drug treatment, or activating downstream signaling pathways that allow tumor cells to bypass signal expression, which leads to drug resistance [144–147]. Tumor cells avoid the toxic of chemotherapy drugs through these resistant methods, Therefore, drug resistance can seriously hinder the treatment of tumors.

The mechanism of drug resistance is complex, several typical resistance mechanisms are as follows:

i) Reduce drug uptake: the heterogeneity of tumor extracellular matrix hinders drug penetration and reduces drug exposure. At the same time, the high interstitial hydraulic pressure of the tumor microenvironment makes the drug accumulate around the tumor cells, making it difficult to enter the tumor cells, thereby gradually inducing drug resistance [148, 149].
ii) Drug-specific target changes: the therapeutic effects of some drugs may be related to specific cellular targets. Mutations in target genes or changes in protein expression may lead to drug resistance [150, 151].
iii) DNA damage repair (DDR): DDR includes direct repair, base excision repair, nucleotide excision repair, DNA mismatch repair and double-strand break damage repair. For DNA-damaging drugs, tumor cells can initiate varieties of different DDR pathways to rapidly repair DNA damage, leading to resistance to such drugs [151, 152].
iv) Heterogeneity: there are differences in gene expression, proliferation and metastatic potential among different tumor cells. When chemotherapy drugs are used on tumor cells, the sensitive cells are killed, while a small number of resistant cells survive and proliferate, resulting in drug resistance [153].
v) Tumor stem cell-like cells (CSCs): CSCs have high tumorigenicity, invasiveness and drug resistance, which are important reasons for the metastasis and recurrence of many malignant tumors [152, 154].
vi) Efflux pumps: the overexpression of energy-dependent drug efflux pumps, such as the drug efflux transporter p-glycoprotein (P-gp), is a well-established mechanism of resistance. P-gp is encoded by the MDR-1 (ABCB1) gene and belongs to a family of energy-dependent transport proteins called ATP-binding cassette (ABC) protein family [155]. These proteins provide energy through ATP to pump chemotherapeutic drugs out of the cell, reducing the accumulation of intracellular drugs and reducing the effective drug concentration, thereby reducing the cytotoxicity of chemotherapeutic drugs to tumor cells [156].

As mentioned above, the mechanism of drug resistance in tumor cells is complex and multiple, and in-depth study is still needed to fully understand it. The mechanisms by which efflux pump proteins, such as P-gp, alter intracellular drug accumulation are best understood. In recent years, the application of nanomaterials in cancer chemotherapy has made great progress, but it is still inevitably affected by drug resistance, and there are still many difficulties in practical application. Nano-engineering with “functional customization” properties, as an efficient bridge between nanomedicine and tumor therapy, can solve the difficulties in its practical application. This review focuses on “increasing the intracellular accumulation of drugs”, and describes the key role of nano-engineering nanomedicines in overcoming tumor resistance.

#### To alter the cellular internalization mechanism of the drug

**Fig. 8:**
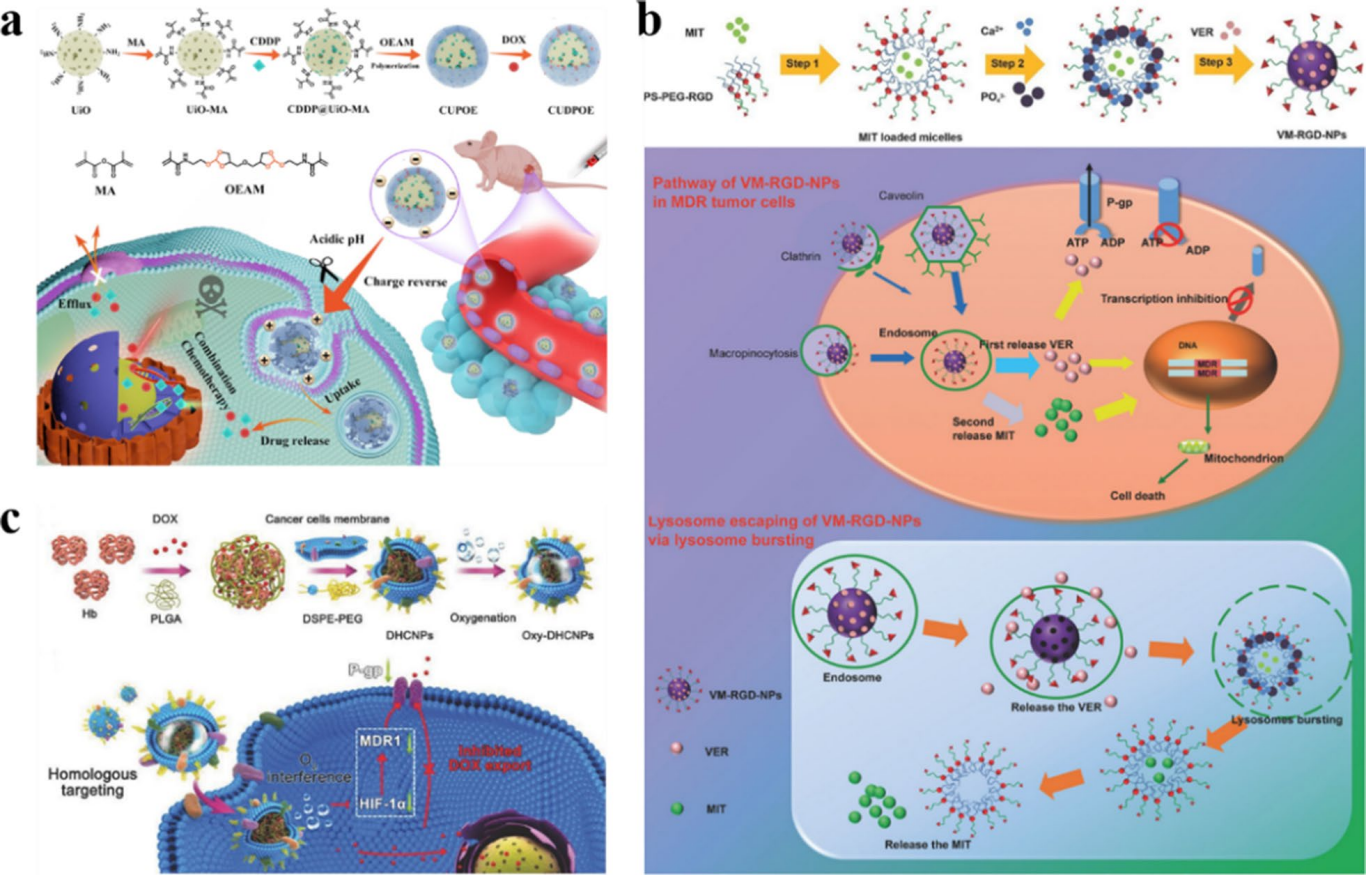
**a** Schematic illustration of the preparation of NPs and the process of combating drug resistance. Reproduced with permission [159]. Copyright 2021, Elsevier. **b** Illustration of the preparation of VM-RGD-NPs and VM-RGD-NPs downregulating P-pg by consuming ATP. Reproduced with permission [156]. Copyright 2018, WILEY-VCH. **c** Preparation process of DHCNPs and schematic diagram of DHCNPs inhibiting drug efflux by regulating HIF-1. Reproduced with permission [171]. Copyright 2017, WILEY-VCH.

Traditional chemotherapy usually uses small molecular weight drugs, most of which diffuse into cells through the cell membrane, while P-gp is mainly distributed in the cell membrane, and the direct passage of drugs through the cell membrane makes it more susceptible to the action of P-gp. Nanoparticles enter cells mainly through endocytosis. Using nanoparticles to load drugs can bypass P-gp, thereby reversing drug resistance and increasing intracellular drug concentration [157, 158]. In addition, based on the unique microenvironment of tumor tissues, nano-engineering nanoparticles can be effectively delivered to the tumor area and promote the uptake of drugs by cells, further increasing the intracellular drug concentration and reversing the drug resistance of cancer cells. Taking advantage of the acidic microenvironment in the tumor area, Hu et al. coated pH-responsive polymer on the surface of MOF for co-delivery of doxorubicin and cisplatin. Nanocarrier maintains the negative surface charge during circulation, which can prolong the blood circulation and ensure stability in vivo. NP then enters tumor cells through endocytosis, and in acidic tumor microenvironment, the outer polymer degrades in response to pH and exposes inner positive MOF, and the positive charge-mediated extravasation further increases cell uptake, so that the drug can accumulate rapidly in the cell and overcome the drug resistance of cancer cells.(Fig. 8a) [159]. Cao et al. designed cationic DOTAP-assisted PEG-PLA NPs for loading Pt (IV) prodrug to bypass P-gp-mediated efflux. And their research shows that PEG can prolong its blood circulation half-life and target the tumor area through the EPR effect, and cationic DOTAP can increase tumor uptake through positive charge-mediated extravasation. Pt (IV) prodrugs can be released and converted into cytotoxic cisplatin under reducing conditions in the cell, thereby effectively increasing the intracellular drug concentration and reversing cisplatin resistance [160]. Therefore, nano-engineering nanoparticles can effectively target tumors, bypass the P-gp on the cell membranpromote cell uptake and increase intracellular drug concentration, thereby reverse tumor chemotherapy resistance.

#### Reduce intracellular ATP levels

Since P-gp is an ATP-dependent protein, reducing the intracellular ATP level can effectively down-regulate P-gp and reduce drug efflux. Wang et al. used the mitochondrial inhibitor ADD to inhibit mitochondrial function, reduce the level of intracellular ATP and down-regulate P-gp, reducing its efflux of DOX in the cell [161]. However, there are some problems in the application of inhibitors in tumor treatment. The biggest obstacle is that the physical and chemical differences between inhibitors and drugs make the two substances unable to co-localize in the tumor area and play a synergistic role. About this conundrum, using engineered nanoparticles as carriers, drugs can be co-delivered and can be efficiently targeted and internalized. At the same time, the release sequence of drug-resistant inhibitors and anti-tumor drugs can also be regulated, giving the inhibitors more time to inhibit drug efflux, thereby obtaining better tumor treatment efficacy. Wang et al. designed a core-shell structured nanoparticle. The drug-resistant inhibitor loaded on the shell is released first, which weakens the efflux pump effect by consuming ATP and downregulating the expression of efflux protein. Then the chemotherapeutic drugs are released to make the anti-cancer drugs more effectively accumulate in the cells to exert their efficacy and overcome the drug resistance of tumor cells [156].

#### Increase intracellular ROS levels

According to the research of Heinrich Sauer’s research group, ROS is a negative regulator of P-gp expression, and increased intracellular ROS level can down-regulate P-gp [162, 163]. In addition, it is well known that dynamic therapy uses sensitizers to induce the production of cytotoxic ROS to treat cancer. Therefore, the combination of sensitizing agents and chemotherapeutic drugs can not only inhibit P-gp expression and overcome drug resistance, but also improve the therapeutic effect on tumors through the synergy of dynamic therapy and chemotherapy. Nano-engineering nanomedicines with customized functions are ideal carriers for combination therapy. Ayman Khdair et al. embedded methylene blue, a photosensitizer that inhibits P-gp, and DOX in AOT-alginate nanoparticles. Methylene blue produces ROS and down-regulate the expression of P-gp, which inhibits anticancer drug efflux and significantly enhances the accumulation of both drugs in cells, greatly improving the efficacy of combined chemotherapy and PDT [155]. Similarly, the cascade drug release platform prepared by Ye et al. utilizes NAD(P)H: quinone oxidoreductase-1 (NQO1) to catalyze β-lapachone to generate ROS, which on the one hand induced the release of cytotoxic DOX from DOX prodrugs, and on the other hand down-regulated P-gp. ATP consumption in the catalytic process will also down-regulate P-gp, overcome drug resistance, and thus exert the best tumor treatment effect [164]. Furthermore, Wu et al. down-regulated the level of GSH, which avoids redox damage, and elevated the intracellular ROS level, thereby down-regulating P-gp expression [165].

#### Increase the local tissue temperature

Heat generated within cells can downregulate P-gp expression or reduce P-gp activity, thereby inhibiting drug outflow [166, 167]. And thermal therapy uses physical methods to heat local or wholebody tissues for a period of time, and uses high temperature to kill tumor cells and treat tumors. Similar to the above, the combination of thermal therapy and chemotherapy can also affect the activity of P-gp, reverse drug resistance, and synergistically promote the therapeutic effect [168]. Lee et al. used Au shell to generate local high temperature in near-infrared light for photothermal treatment. After photothermal treatment, the efflux of drugs is significantly inhibited, and the accumulation of DOX in the cells is increased. The synergy of photothermal and chemotherapy makes the therapeutic effect significantly enhanced [166]. Deng et al. used polymeric micelles with an upper critical solution temperature (UCST) to load photothermal agents and DOX. Their study shows that the photothermal effect of the photothermal agent can completely dissociate the nanocarrier and fully release the drug, and the photothermal effect reduces the expression of P-gp by 72%, avoiding drug efflux, synergistically reversing tumor drug resistance, and greatly improving the therapeutic effect of tumors [169].

#### Alleviating tumor hypoxic microenvironment

The inherent hypoxia properties of the tumor microenvironment make the hypoxia-inducible factor HIF-1 overexpression, which participates in the invasion and metastasis of cancer cells, and induces the overexpression of P-gp, resulting in chemotherapy resistance [170]. Normalizing the partial pressure of oxygen in the tumor area can degrade HIF-1α and avoid its effects. Tian et al. used nanoparticles functionalized by cancer cell membranes to load hemoglobin and DOX. Highly selective delivery of DOX and oxygen to homologous tumor cells through a homologous binding mechanism. Targeted oxygen supply overcomes tumor hypoxia, inhibits HIF-1α and its induced P-gp expression, so that DOX can effectively accumulate in tumors and overcome hypoxia-induced chemotherapy resistance (Fig. 8c) [171]. Zan et al. used peptide-modified nanoparticles to load drugs that relieve hypoxia and chemotherapy. Polypeptides can target tumors and penetrate cell membranes, allowing drugs to accumulate in tumor cells, and then reprogram the tumor hypoxic microenvironment, thereby down-regulating HIF-1a and inhibiting tumor cell resistance [172]. Chen et al. designed core–shell CuO@ZrO_2_ nanocomposites loaded with the chemotherapy drug DOX, which can produce large amounts of O_2_ under MW irradiation, significantly alleviating tumor hypoxia and overcoming chemotherapy resistance. The tumor suppression rate of the nanocomposites (92.14%) was significantly higher than that of nanocomposites could not produce oxygen (51.11%), which significantly improved the effect of MW therapy and chemotherapy [173]. Subsequently, their team designed another CuO-based NPs that can continuously release O_2_ under MW irradiation, downregulate HIF-1α, and overcome radiotherapy resistance [174].

#### Gene silencing

Silencing the expression of efflux protein at gene level is also an effective method to reverse drug resistance. Small interfering RNA(siRNA) is a research hotspot at present. However, siRNA lacks selective targeting ability, and its physicochemical properties are diverse from those of small molecule drugs, leading to differences in biodistribution and tumor accumulation, which seriously affect its therapeutic effect. Nanoengineered nanoparticles can solve this conundrum. For example, Yogesh B. Patil et al. used PLGA-PEI nanoparticles coloaded with siRNA and paclitaxel, and biotin was modified on the surface of the nanoparticles to target tumors. The expression of P-gp can be inhibited by silencing the MDR1 gene encoding P-gp by siRNA interference, which can improve the sensitivity of tumor cells to drugs and effectively overcome drug resistance [175].

#### Other methods

The existence of CSCs is an important reason of metastasis and recurrence of many malignant tumors, killing CSCs can effectively inhibit tumor growth and prevent postoperative tumor recurrence and metastasis. Shen et al. designed a sequential release nano-delivery carrier. The drug ATRA that induces stem cell differentiation is physically encapsulated in the nano-carrier, while the chemotherapeutic drug CPT is assembled into nanoparticles through the coupling of ROS unstable oxalate bonds with polymers. After being taken up by cancer stem cells, the nanoparticles trigger the rapid release of ATRA through hypoxia, which induces stem cell differentiation. When stem cells differentiate, the intracellular ROS increases with the production of mitochondrial superoxide, and the oxalate bond is broken in response to ROS to release the chemotherapeutic CPT (Fig. 8b) [176]. Nanoparticles regulated by nano-engineering enable controlled and orderly release of drugs in cancer stem cells, reducing stem cell-related drug resistance and enhancing the effect of chemotherapy. Therefore, nanocarriers designed by nano-engineering for co-loading drugs and inhibitors provide a rational approach to overcome drug resistance in tumor cells.

Epithelial-mesenchymal transition (EMT) is the key process that promotes tumor invasion and metastasis, and the acquired drug resistance of tumor cells is largely attributed to EMT [152, 177]. Therefore, drug resistance of tumor cells can also be effectively overcome by preventing EMT. Guo et al. designed ADH-1-HA-MTN nanocarriers loaded with DOX to target tumors by specific recognition between HA and CD44. ADH-1 prevents the epithelial-mesenchymal transition process by re ducing the expression of N-cadherin, while TiO_2_ generates ROS, which combine with DOX accumulated in tumor cells to kill tumor cells. Their results proved that drug resistance in tumor cells can also be effectively overcome by preventing epithelial-mesenchymal transition [178].

All in all, the progress of nano-engineering provides new ideas for overcoming tumor drug resistance. Nano-engineering nanoparticles can help chemotherapeutic drugs bypass P-gp on the cell membrane and enter tumor cells, and can inhibit the expression or activity of P-gp through ATP, ROS, heat, HIF-1α, gene and other pathways to avoid drug efflux, thereby reversing drug resistance, which is the key to further improve the therapeutic effect.

### Immunosuppressive

In the process of tumor treatment, if the patient’s own immune system can synergize with chemotherapy, radiotherapy, phototherapy and other treatment methods, it can kill the primary tumor while establishing an immune memory effect in the body and inhibit tumor recurrence and metastasis. However, the complex microenvironment of the tumor area has highly immunosuppressive effect, which seriously affects the therapeutic effect of the tumor.

Immunosuppression refers to the inhibitory effect on the immune response, which can be attributed to immunosuppressive cells, immunosuppressive cytokines, immune checkpoints and the loss of immunogenicity of tumor cells [14]. The main immunosuppressive cells are tumor-associated macrophages (TAMs), Myeloid-Derived Suppressor Cells (MDSCs) and Regulatory T cells (Tregs) [13, 179–181]. Activated macrophages have two phenotypes: the M1 phenotype that can effectively kill tumor cells and the M2 phenotype that promotes tumor growth. M1 and M2 macrophages can be transformed into each other. Mainly M2 phenotype in the tumor microenvironment, which promotes tumor growth and leads to immunosuppression [179, 182]. MDSCs are heterogeneous cells produced under pathological conditions such as cancer and inflammation, which can significantly inhibit T cell responses, especially the function of tumor-infiltrating CD8^+^ T cells [180, 183]. Tregs can inhibit the activation and function of other immune cells. These immunosuppressive cells and tumor cells secrete various immunosuppressive cytokines, such as transforming growth factor-β (TGF-β), vascular endothelial growth factor A (VEGF-A) and interleukin-10 (IL-10), these factors constitute an immunosuppressive tumor microenvironment. At the same time, the inherent hypoxia and high interstitial hydraulic pressure of the tumor microenvironment will further aggravate immunosuppression [184, 185]. In addition, tumor cells hijacked immune checkpoints (such as programmed cell death 1/programmed cell death ligand 1 (PD-1/PD-L1), cytotoxic T lymphocyte antigen 4 (CTLA- 4)) that can regulate their own immune function and prevent autoimmunity, thereby inhibiting T cell responses, making them unable to activate and kill tumor cells.

The serious adverse effect of tumor immunosuppression status on tumor treatment has led people to seek various methods to solve this problem. The rapid development of nano-engineering may provide a powerful boost for it. This section will briefly introduce the methods of alleviating tumor immunosuppression and the application of nano-engineering in these methods.

#### Drugs directly inhibit immunosuppressive cells

**Fig. 9:**
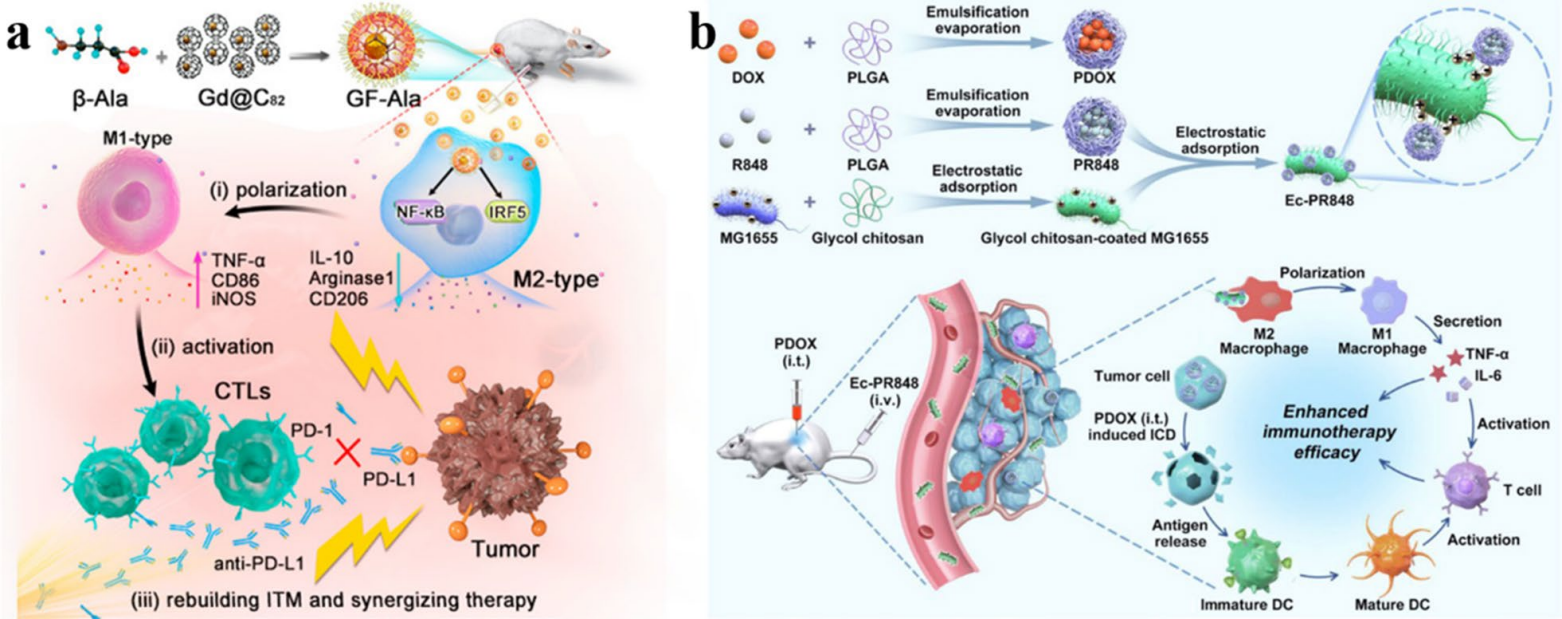
**a** Schematic illustration of the preparation of GE-Ala and its effect mechanism in vivo. Reproduced with permission [186]. Copyright 2020, American Chemical Society. **b** Schematic illustration of the preparation of Ec-PR848 NPs and ICD enhanced the efficacy of immunotherapy based on TAMs polarization. Reproduced with permission [190]. Copyright 2021, American Chemical Society

Macrophages are the most important immunosuppressive cells in the tumor microenvironment. By stimulating the M2 phenotype macrophages to polarize to the M1 phenotype, they can reshape the immunosuppressive microenvironment, reactivate the immune response, and effectively kill the tumor. There are drugs that can reprogram M2 macrophages into M1 macrophages, and the use of engineered nanoparticles to deliver drugs can greatly improve the safety and effectiveness of treatment. The drug can accumulate in the tumor site to effectively relieve immune suppression, while avoiding reprogramming TAMs in normal tissues and destroying the normal immune system in the body. Li et al. modified Gd@C_82_ with β-alanine to obtain GF-Ala nanoparticles, after GF-Ala NP is internalized by TAMs, it can activate the NF-κB and IRF5 pathways, reprogram TAMs from the tumor-promoting M2 phenotype to the tumor-killing M1 phenotype, and increase the infiltration of CD8^+^ cytotoxic T lymphocytes (CTLs), thereby performing effective immunotherapy and inhibiting tumor growth (Fig. 9a) [186]. In the same way, depletion of MDSCs and Treg in the tumor microenvironment can also improve tumor immunosuppression. Kalliopi Domvri et al. used CuS-NC to wrap the Norvaline/Sunitinib (NorSun) prodrug complex, and then used PLGA-PEG for surface modification. PLGA-PEG is used to protect CuS-NC and prolong the blood circulation half-life, allowing NPs to accumulate in tumors through the EPR effect. Afterwards, CuS/NorSun can down-regulate MDSC and inhibit Treg, while activating NK cells and antigen-specific T cell responses [183]. Sun et al. used ROS-responsive nanoparticles to co-deliver photosensitizers and sorafenib. Under light, the photosensitizer generates ROS for PDT and degrades ROS-responsive nanoparticles to release sorafenib. Sorafenib not only kills tumors, but also inhibits MDSC and Treg, enhances the function of tumor-specific CTLs, and indirectly enhances the efficacy of PDT [187]. Therefore, nano-engineering nanomedicines targeting immunosuppressive cells can effectively alleviate tumor immunosuppression and improve tumor treatment effects.

#### Induce the immunogenic cell death of tumor cells

The loss of immunogenicity of tumor cells is one of the reasons for immunosuppression. The use of inducers to treat tumor cells may trigger immunogenic cell death (ICD) and increase the immunogenicity of tumor cells. ICD releases tumor-associated antigens (TAA) and damage-associated molecular patterns (DAMPs), which promote the maturation of dendritic cells (DC). After that, the DC presents tumor antigens to T cells to promote the recruitment and activation of CTLs, thereby inhibiting tumor growth. Nano-engineering nanomedicines can be used as an inducer to directly or indirectly trigger ICD. Wen et al. prepared Cu_2 − x_Te NPs with glutathione oxidase (GSHOx) and peroxidase (POD) activities, which synergistically produce oxidative stress by consuming GSH and producing ROS. Oxidative stress can induce ICD to eliminate primary tumors, and can polarize M2 phenotype TAMs to M1 phenotype, promote the secretion of inflammatory cytokines (TNF-a and IL-6), activate T cell-based immune response, while building immune memory to inhibit tumor metastasis and recurrence [188].

Engineered nanoparticles can not only effectively target the tumor area and achieve the controlled release of the inducer, but also co-deliver drugs with different physical and chemical properties, and make it work synergistically. Qi et al. designed a pH- and heat-sensitive nanoparticles loaded with LfcinB. Nanoparticles can target tumors through the EPR effect, and controllable release of drugs in response to acid (pH 5.5) and heat (43 °C) under lysosome and microwave irradiation. LfcinB destroys the mitochondrial membrane to induce tumor cell apoptosis, and induces ICD, promotes the proliferation of CD4^+^ and CD8^+^ T cells and the secretion of cytokines, and enhances the immune response [189]. Wei et al. used engineered E. coli to co-deliver Resiquimod (R848) and pDOX with different physicochemical properties. E. coli and R848 combined to polarize the M2 macrophages to M1 macrophages; pDOX triggers ICD to activate CTLs, further alleviating the tumor immunosuppression (Fig. 9b) [190]. Similarly, Zhao et al. co-delivered PTX and the immunostimulant interleukin-2 (IL-2). PTX induces ICD, enhances tumor immunogenicity, and relieves immunosuppression; IL-2 promotes the anti-tumor immune response of immune effector cells [191]. Therefore, ICD can induce the tumor microenvironment to change from an immunosuppressive state to an immunostimulatory state, and enhance the immune response.

#### Restrict immune checkpoints

**Fig. 10:**
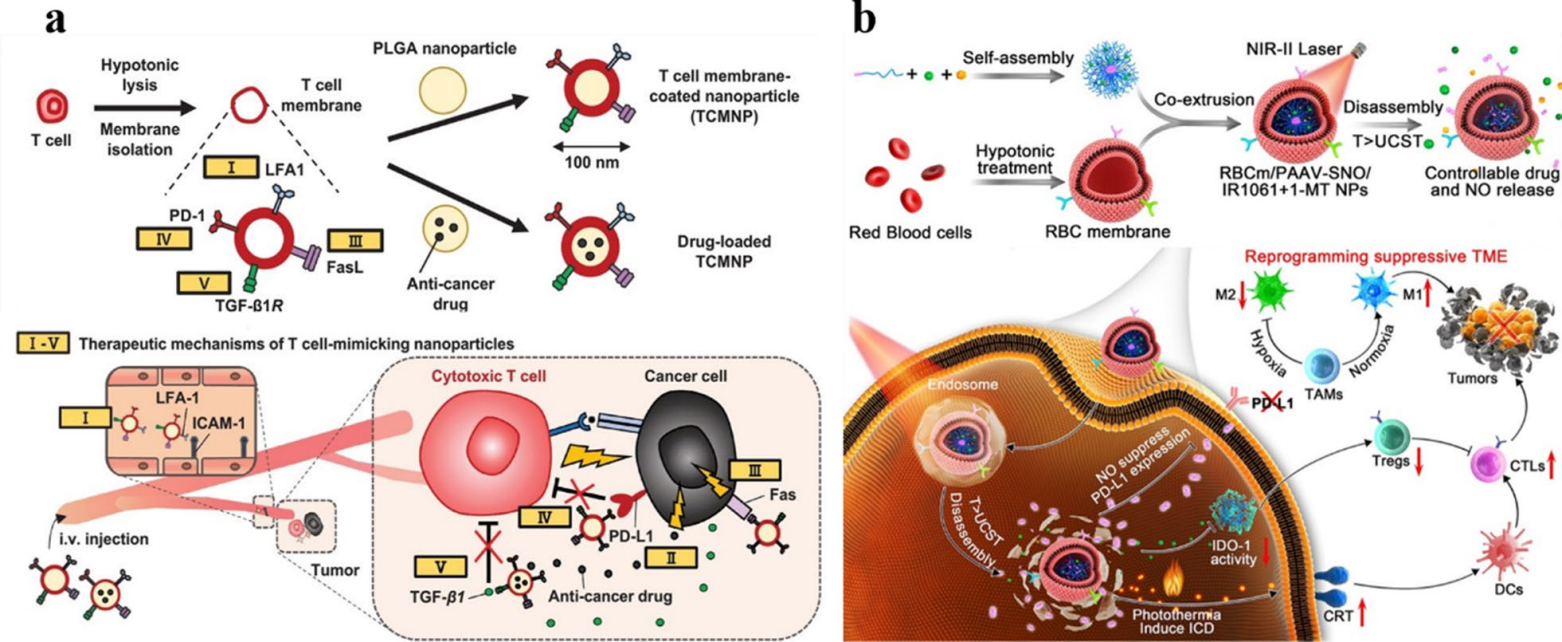
**a** Schematic illustration of the preparation of TCMNPs and the mechanism of treatment by immunosuppressive checkpoints. Reproduced with permission [194]. Copyright 2020, Wiley-VCH. **b** Schematic illustration of the preparation and releasing process of RBCm-coated nano-bullets and mechanism of reprogramming tumor immunosuppressive microenvironment. Reproduced with permission [199]. Copyright 2020, American Chemical Society

Immune checkpoint is a co-stimulatory and inhibitory signal that regulates antigen recognition in the process of immune response. Inhibitors of immune checkpoints (such as PD-1, PD-L1 and CTLA-4) play an important role in alleviating tumor immunosuppression. However, immune checkpoint inhibitors still have problems such as poor stability and poor targeting. In addition, the efficacy of immune checkpoint inhibitors themselves is also affected by the tumor immunosuppressive microenvironment, and needs to be coordinated with other methods to alleviate immunosuppression. Engineered nanoparticles can safely and effectively deliver immune checkpoint inhibitors and other therapeutic agents to the tumor area, so that they can work synergistically. Yu et al. used RBCm-coated nanoparticles to load photosensitizer, ROS reactive prodrug PXTK and anti-PD-L1 peptide dPPA for chemotherapy, PDT and immunotherapy. RBCm coating can prolong the blood circulation half-life, enhance targeting and promote cell uptake. Under light, the photosensitizer PheoA quickly generates ROS for PDT, and degrades PXTK to release PTX. The anti-PD-L1 peptide dPPA can effectively block the combination of PD-1 and PD-L1, and promote the proliferation of CD4^+^ T cells and the secretion of IL-12. In addition, PDT can induce ICD, promote the proliferation of CTLs and the secretion of TNF-α. The synergistic effect of ICD and immune checkpoints can relieve immune suppression [192]. Wang et al. designed MMP-2 sensitive nanoparticles to deliver aPD-L1 and photosensitizer ICG to the tumor area. Nanoparticles release aPD-L1 and ICG in response to the high expression of MMP-2 at the tumor site. aPD-L1 blocks the combination of PD-1 and PD-L1, and ICG generates ROS for PDT to directly kill tumor cells, and induces ICD to enhance immune response [193]. Kang et al. used T cell membranes to coat PLGA NPs to obtain TCMNPs. The proteins on the T cell membranes can exert a variety of functions. Their research shows that TCMNPs can actively target tumors through membrane proteins and induce FasL-dependent apoptosis to kill cancer cells; PD-1 on the cell membrane can be used to competitively bind with PD-L1 on the cancer cell membrane to reactivate CTLs; TGF-β1R on the cell membrane can also be used to eliminate the immunosuppressive molecule TGF-β1 and restore the cytotoxicity of CTLs (Fig. 10a). Therefore, TCMNPs can effectively relieve immunosuppression and enhance immune response [194].

#### Adjust external environmental factors

Hypoxia is an inherent feature of the tumor microenvironment, and studies have shown that hypoxia can enhance the immunosuppressive effect. First, hypoxia promotes the differentiation of TAMs into immunosuppressive M2 phenotype and promotes the accumulation of Tregs; second, tumor cells overexpress PD-L1 under hypoxic conditions; finally, hypoxia can interfere the secretion of cytokines [195, 196]. These factors work together to cause stronger immunosuppressive effect. Therefore, alleviating the hypoxia of the tumor can greatly improve the immunosuppression. There are already many nano-engineering nanomedicines to alleviate hypoxia.

First, oxygen can be directly transported to the tumor area through oxygen-carrying nanomaterials, such as perfluorocarbons. Liquid perfluorohexane (PFH) can dissolve a large amount of oxygen as the oxygen carrier [197]. This method usually has low oxygen load and poor biocompatibility. Therefore, a more effective method for alleviating tumor hypoxia has been developed-generating oxygen in situ in the tumor area. For example, the use of chemical substances to react with endogenous H_2_O_2_ or the use of catalysts to convert endogenous H_2_O_2_ into oxygen in situ [198]. This method also has problems. The endogenous H_2_O_2_ is limited, so exogenous H_2_O_2_ can be delivered to the tumor area, and then it can catalyze the decomposition of H_2_O_2_ and promote the production of oxygen. Song et al. used PEG-modified Liposome to load catalase (CAT@liposome) and H_2_O_2_ (H_2_O_2_@liposome), respectively, and deliver them to the tumor area to make the oxygen concentration continue and moderately increase and relieve tumor hypoxia. Their research shows that alleviating hypoxia can polarize M2 macrophages into M1 macrophages, reversing the immunosuppressive TME. Combined with CTLA-4 checkpoint blockade, it can effectively inhibit Tregs, promote the infiltration of CTLs, and further inhibit tumor progression [184]. In addition, normalizing tumor blood vessels or dilating tumor blood vessels through mild hyperthermia, thereby increasing blood flow, can also effectively alleviate hypoxia. Yang et al. used the heat-sensitive nitric oxide (NO) donor PAAV-SNO to self-assemble into nanoparticles and co-loaded photothermal agents and drugs (1-MT), and then coated them with RBCm. RBCm-coated nanoparticles can be targeted to the tumor area. Then, under the irradiation of the light, the photothermal agent performs photothermal conversion for local hyperthermia. The photothermal therapy simultaneously induces ICD, promotes the increase of CD8^+^ CTLs at the tumor site, and stimulates the immune response. Higher temperature can also promote the decomposition of PAAV-SNO copolymer and release NO and 1-MT accurately at the tumor site. 1-MT can inhibit the activity of IDO-1 to restore CD8^+^ CTLs function and inhibit the proliferation of Tregs. NO can induce the expression of endogenous angiogenic factors, normalize tumor blood vessels, and cooperate with the expansion of tumor blood vessels caused by mild hyperthermia to reduce hypoxia, significantly reduce hypoxia-induced PD-L1 expression, and reshape the immunosuppressive microenvironment (Fig. 10b) [199].

High interstitial hydraulic pressure is also one of the characteristics of the tumor microenvironment. Normally, high accumulation of hyaluronan (HA) in the tumor microenvironment leads to an increase in the interstitial pressure. Studies have shown that high molecular weight HA can inhibit the polarization of M1 macrophages, enhance the polarization of M2 macrophages, and induce immunosuppression [200, 201]. Therefore, by decomposing high-molecular-weight (HMW) HA into low-molecular-weight (LMW) HA, immunosuppression can also be effectively alleviated. Feng et al. prepared engineered PH20-exosomes and modified them with FA. Surface-modified FA can not only improve the targeting ability, but also inhibit the migration of tumor cells caused by hyaluronidase through the FA receptor/cSrc-signaling pathway. Hyaluronidase PH20 can degrade HMW-HA into LMW-HA, polarize tumor-promoting M2-like TAMs into anti-tumor M1-like TAMs, and relieve immunosuppression [185].

#### Anti-immunosuppressive factors at the genetic level

**Fig. 11:**
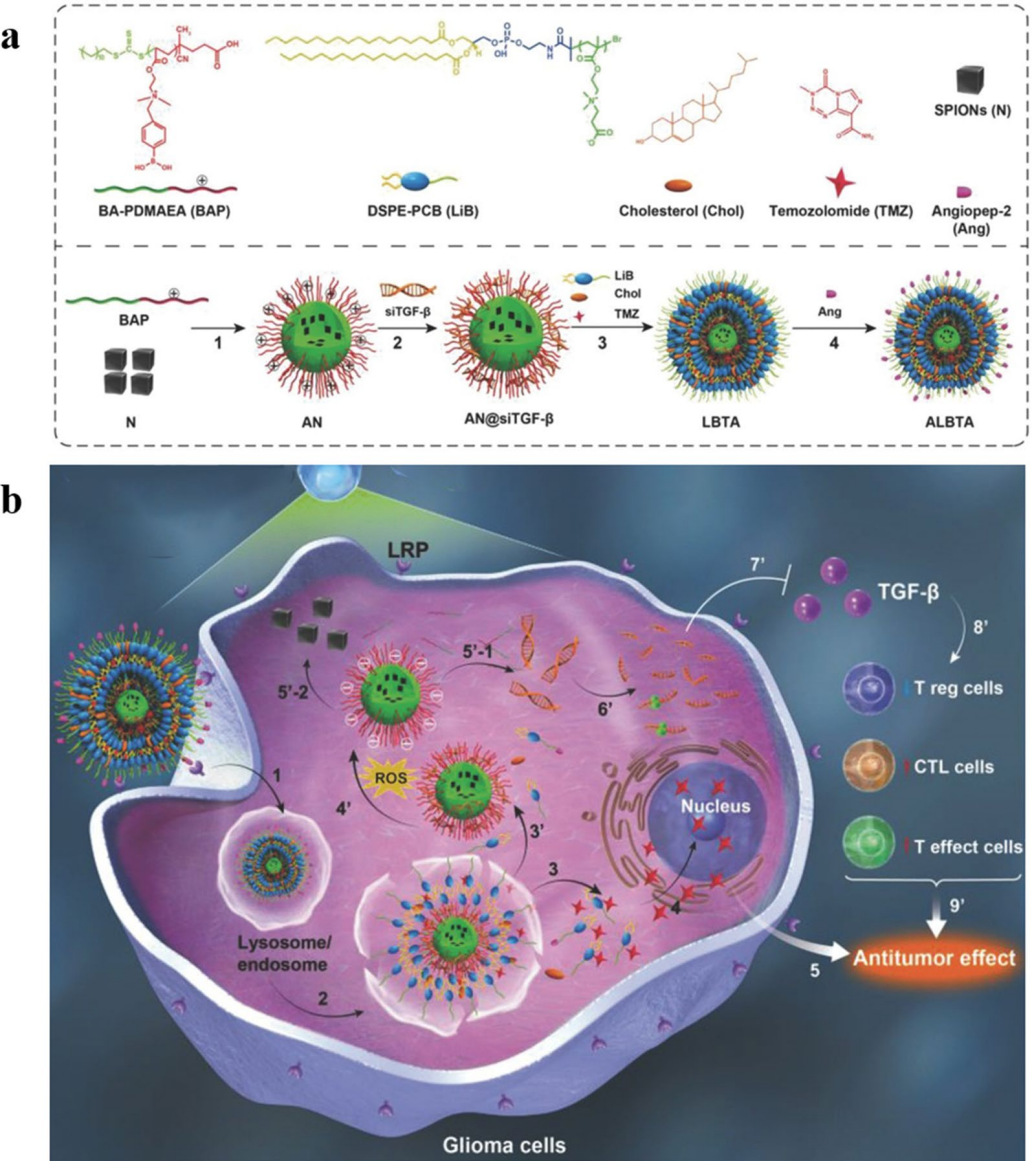
Schematic illustration of **a** the preparation of targeted nanoparticles and **b** the mechanism of cellular uptake, subcellular drug delivery, and relieving immunosuppression. Reproduced with permission [206]. Copyright 2018, WILEY-VCH.

RNA interference (RNA interference, RNAi) can specifically regulate gene expression and open up new ways for gene therapy, which is one of the research hotspots. Using small interfering RNA (siRNA) and double-stranded RNA (dsRNA), etc., can directly silence the expression of immunosuppressive factors at the gene level, thereby improving immunosuppression. However, the lack of selective targeting ability, poor membrane penetration, and poor stability of siRNA and dsRNA limit their therapeutic effects. The progress of nano-engineering provides more favorable conditions for RNAi. Nanoparticles can effectively load siRNA or dsRNA, protect them, and target them to the tumor area. Nano-engineering nanoparticles can also optimize targeting performance, promote cell internalization, or controllable release in response to stimuli. Therefore, engineered nanoparticles can effectively improve the safety and effectiveness of RNAi. In addition, engineered nanoparticles can also be co-loaded with other therapeutic agents to synergistically alleviate immune suppression, activate immune responses, and effectively treat tumors. Indoleamine 2,3-dioxygenase-1 (IDO1) can affect the activity of DCs, increase the number of Tregs, and induce apoptosis of CTLs, resulting in immunosuppression. Huang et al. used cationic lipid-assisted nanoparticles (CLANs) to coat small interfering RNA (siIDO1) targeting IDO1. siIDO1 significantly down-regulates IDO1 in tumor tissues, alleviating immunosuppression [202]. Maryam Esmaily used chitosan-lactic acid (CL) NPs to load anti-CTLA-4 siRNA molecules [203], Ali Masjedi et al. modified PEG on the surface of CL NPs, and then loaded A2aR specific siRNA [204], which significantly inhibit the expression of corresponding CTLA-4 or A2aR, thereby alleviating immunosuppression and enhancing the immune response. Manisit Das et al. conjugated AEAA ligands on the surface of nanoparticles loaded with RIG-I agonist and Bcl2 gene silencing agent (ppp-dsRNA) to further improve targeting ability and promote internalization. ppp-dsRNA effectively relieves immunosuppression, promotes immune response and silences the anti-apoptotic gene Bcl_2_[205]. Qiao et al. used dual-targeting and ROS-responsive nanocarriers to deliver siTGF-β and the drug TMZ. Through the dual targeting peptide angiopep-2 on the surface of the nanoparticle, it crosses the blood-brain barrier and actively targets tumor cells. After the nanoparticles are internalized into the cell, they are protonated in the acidic environment of the lysosome to promote the lysosomes escape. Then the nanoparticles release siTGF-β in response to ROS, and siTGF-β down-regulates TGF-β to regulate T cells, improve the tumor immune microenvironment, and improve the efficacy of TMZ (Fig. 11). Synergistic anti-tumor therapy significantly prolonged the survival time of mice [206].

In short, immunosuppressive cells, immunosuppressive cytokines, immunosuppressive checkpoints, and the loss of immunogenicity of tumor cells together make the tumor in an immunosuppressive state, inhibiting the immune response of the immune system to cancer cells, which is a major obstacle to tumor therapy. Nano-engineering can endow nanoparticles with the desired functions, enabling them to cooperates with various methods to deal with immunosuppression, which can polarize M2 macrophages into M1 macrophages and inhibit the proliferation of immunosuppressive cells such as MDSCs and Treg, inhibit the secretion of immunosuppressive cytokines such as TGF-β and IL-10, and promote the secretion of inflammatory cytokines such as TNF-α and IL-12, activate CD4^+^, CD8^+^ T cells and NK cells and other immune cells, thereby alleviating immune suppression, enhancing immune response, and obtaining better tumor treatment effects.

## Conclusion and outlook

In this review, we summarized nano-engineering and their vital applications in tumor treatment. Nano-engineering is to tune the performance of nanomedicines from manipulating the size, surface properties and structure. Nano-engineering can regulate the dispersibility, stability, biocompatibility, and surface reactivity of nanomedicines for better circulation, distribution, and excretion in the body. Furthermore, building blocks with different excellent properties can be combined according to requirements to customize different composite functions and improve the overall performance of nanomedicines. Therefore, nano-engineering nanomedicines with customized functions can overcome the problems in tumor treatment, including the poor delivery, tumor cell drug resistance, and the immunosuppressive microenvironment, thereby improving tumor treatment effects.

Nano-engineering can endow nanomedicines with desired functions, leading to diversified means of cancer therapies and diagnosis, which are expected to alleviate more challenges in cancer treatment, and introduce fresh research and clinical application prospects. However, as an emerging technology, it still has many limitations. To further expand its application value, nano-engineering nanomedicines still need to be advanced to meet the urgent needs of tumor treatment and other fields. We call for deeper research from the following three aspects:

1. The biggest advantage of nano-engineering is that it can customize the function of nanomedicines. However, there is no clear understanding of the relationship between the structure and performance of nanomedicines due to the complexity and diversity. Synthesizing nanomedicines with desired properties is still determined by trial and error, wasting time and resources, and the intended goal of the design is not necessarily the same as the end result. Therefore, its combination with artificial intelligence or machine learning is crucial. Using the powerful data processing ability to combine the experimental results of the existing research may determine the influence of the raw materials and synthesis methods on the size, morphology, structure, etc. of the nanomedicines, and then further confirm the relationship between them and the final performance, which can be used to design or predict nanomedicines, and accurately obtain nanomedicines with desired properties.

(2) Nanomedicines can be easily combined with smart response based on the “customized” properties of nano-engineering to design and prepare tumor microenvironment smart-responsive nanosystem. Smart-responsive nanomedicines can be activated by endogenous or exogenous stimuli, such as pH, enzymes, GSH, ROS, ATP, light, ultrasound, microwave, etc., for the controlled drug release, biosensing, and tumor therapy. At present, there have been many on smart nano-drug carriers, while the research on in vivo smart response imaging and therapy still needs to be further developed. At the same time, compared with single response system, nanomedicines that simultaneously respond to external and internal stimuli are more accurate and effective in tumor treatment. However, the more complex the system, the more difficult it is to design and synthesize, which requires a deep understanding of the multi-fields of physics, chemistry, biology, medicine, etc.

(C) The biocompatibility of nanomedicines is crucial. Although nanomedicines will be tested for safety before practical application, the long-term toxicity in body is still a major obstacle, including but not limited to the accumulation of non-degradable nanomedicines and the caused damage to the human body. Through nano-engineering, the size, morphology, surface properties, structure, etc. of nanomedicines can be optimized, but it is mainly focused on modifying the surface, and the toxicity of the material itself has not been reduced. It is necessary to further seek out the factors affecting the biosafety of nanomedicines, and establish a feasible long-term tracking and evaluation system.

In conclusion, nano-engineering has achieved encouraging results, but there is still much room for progress in its practical application. With the continuous deepening of interdisciplinary cooperation, nano-engineering can endow nanomedicines with more ideal functions. It is believed that nano-engineering will play a greater role in biomedical field, including but not limited to the applications of nano-engineering nanomedicines in cancer therapy.
